# PCP Signaling between Migrating Neurons and their Planar-Polarized Neuroepithelial Environment Controls Filopodial Dynamics and Directional Migration

**DOI:** 10.1371/journal.pgen.1005934

**Published:** 2016-03-18

**Authors:** Crystal F. Davey, Andrew W. Mathewson, Cecilia B. Moens

**Affiliations:** Division of Basic Science, Fred Hutchinson Cancer Research Center, and University of Washington Molecular and Cellular Biology Graduate Program, Seattle, Washington, United States of America; University of Virginia Health System, UNITED STATES

## Abstract

The planar cell polarity (PCP) pathway is a cell-contact mediated mechanism for transmitting polarity information between neighboring cells. PCP “core components” (Vangl, Fz, Pk, Dsh, and Celsr) are essential for a number of cell migratory events including the posterior migration of facial branchiomotor neurons (FBMNs) in the plane of the hindbrain neuroepithelium in zebrafish and mice. While the mechanism by which PCP signaling polarizes static epithelial cells is well understood, how PCP signaling controls highly dynamic processes like neuronal migration remains an important outstanding question given that PCP components have been implicated in a range of directed cell movements, particularly during vertebrate development. Here, by systematically disrupting PCP signaling in a rhombomere-restricted manner we show that PCP signaling is required both within FBMNs and the hindbrain rhombomere 4 environment at the time when they initiate their migration. Correspondingly, we demonstrate planar polarized localization of PCP core components Vangl2 and Fzd3a in the hindbrain neuroepithelium, and transient localization of Vangl2 at the tips of retracting FBMN filopodia. Using high-resolution timelapse imaging of FBMNs in genetic chimeras we uncover opposing cell-autonomous and non-cell-autonomous functions for Fzd3a and Vangl2 in regulating FBMN protrusive activity. Within FBMNs, Fzd3a is required to stabilize filopodia while Vangl2 has an antagonistic, destabilizing role. However, in the migratory environment Fzd3a acts to destabilize FBMN filopodia while Vangl2 has a stabilizing role. Together, our findings suggest a model in which PCP signaling between the planar polarized neuroepithelial environment and FBMNs directs migration by the selective stabilization of FBMN filopodia.

## Author Summary

Planar cell polarity (PCP) is a common feature of many animal tissues. This type of polarity is most obvious in cells that are organized into epithelial sheets, where PCP signaling components act to orient cells in the plane of the tissue. Although, PCP is best understood for its function in polarizing stable epithelia, PCP is also required for the dynamic process of cell migration in animal development and disease. The goal of this study was to determine how PCP functions to control cell migration. We used the migration of facial branchiomotor neurons in the zebrafish hindbrain, which requires almost the entire suite of PCP core components, to address this question. We present evidence that PCP signaling within migrating neurons, and between migrating neurons and cells of their migratory environment promote migration by regulating filopodial dynamics. Our results suggest that broadly conserved interactions between PCP components control the cytoskeleton in motile cells and non-motile epithelia alike.

## Introduction

The Planar Cell Polarity (PCP) signaling pathway is best understood as a cell contact dependent mechanism for generating and maintaining polarity in the plane of an epithelium [1, 2]. Its function was first described in the static epithelial cells of the fly where the molecular asymmetry of “core” PCP proteins results in the morphological asymmetry of a single actin-rich hair at the distal side of each wing cell [3–5]. Subsequently, planar polarity established by the core pathway has been shown to be a characteristic of many epithelial tissues in vertebrates and invertebrates alike [6–10]. The core PCP pathway is comprised of two protein complexes that localize to distinct cell membranes. In the fly wing, the transmembrane protein Frizzled (Fz) is confined to distal apical cell junctions along with the cytosolic proteins Disheveled (Dsh) and Diego (Dgo), while the transmembrane protein Van Gogh (Vang) (Strabismus(Stbm)) and the cytosolic protein Prickle (Pk) are proximally localized. This molecular asymmetry of PCP promotes actin polymerization at the distal side of the cell, downstream of Fz and Dsh [11–13]. While the factors that initially polarize PCP components are context dependent [14], the asymmetric localization of PCP proteins is maintained within polarized cells via intracellular destabilizing interactions between the Vang complex and the Fz complex [15, 16]. This polarization of PCP proteins is coordinated between cells by the formation of intercellular stabilizing interactions between Vang and Fz complexes across cell junctions [17–21]. In spite of the antagonistic roles of Vang and Fz complexes, loss of function of any core PCP component results in a loss of polarity.

While PCP is well known for its role in stable epithelia [22–24], core PCP components have also been implicated in dynamic cellular processes such as cell migration. How PCP controls directed cell movements is best, though incompletely, understood in coherently migrating cells such as those undergoing convergent extension [25–37]. However, independently migrating cells also require PCP [38–44]. Here, as our model we use the stereotyped and conserved migration of cranial motor neurons in the vertebrate hindbrain [45–47]. This enabled us to study *in vivo* how PCP can regulate the migration of non-coherent cells and to determine how PCP signaling between different cell types, the migrating neurons and the cells through which they migrate, can modulate migratory cell behaviors.

The PCP pathway drives the stereotyped tangential migration of facial branchiomotor neurons (FBMNs) in the vertebrate hindbrain. FBMNs are a subset of cranial branchiomotor neurons that originate ventrally in rhombomere (r)4 and undergo a posterior migration to r6 where they form the facial motor nucleus, whose axons exit the hindbrain in r4 and innervate muscles derived from the second branchial arch [45, 47]. Forward genetic screens in the zebrafish have identified multiple core PCP components (Vangl2, Pk1b, Fzd3a, Celsr2 and Scribble) as being required for FBMN migration [31, 48–51]; this PCP requirement has also been shown for mouse FBMN migration [52–54]. Unlike the cell migrations mentioned above, screens have failed to identify a role for Wnts or other chemotactic cues. Although it is clear that many components of the PCP pathway are required for tangential FBMN migration, how these components regulate this highly dynamic process is unknown.

As a first step in answering this question we defined the cell types participating in PCP signaling during FBMN migration, as previous studies using a range of approaches have yielded conflicting results [31, 48, 49, 51, 55]. Using the Gal4/UAS system to systematically disrupt PCP in a cell-type and rhombomere-specific manner, we demonstrate the dual requirement for PCP within FBMNs and the planar-polarized r4 neuroepithelial environment in which they arise, and identify reciprocal PCP-dependent interactions between FBMNs and the planar-polarized floorplate as being sufficient, though not required, to promote migration. Since cell migration results from the contact-dependent stabilization of cellular protrusions and PCP signaling is known to regulate actin dynamics, we examined the protrusive activity of single FBMNs using high-resolution single-cell time-lapse microscopy in chimeric embryos and demonstrate opposing functions for the PCP core components Fzd3a and Vangl2 in regulating FBMN filopodial protrusive activity *in vivo*. Within FBMNs we show that Fzd3a is required to stabilize filopodia while Vangl2 has an antagonistic, destabilizing role. However, in the migratory environment we show that Fzd3a is required to destabilize filopodia while Vangl2 has a stabilizing role. In spite of having antagonistic roles at the cellular level, Vangl2 and Fzd3a mutants have the same FBMN migration phenotype. These findings are thus reminiscent of the intracellular antagonistic versus intercellular stabilizing roles that core PCP proteins perform in stably polarized epithelia. Consistent with a role for Vangl2 in regulating filopodial dynamics, we show that Vangl2 localizes transiently to the tips of retracting FBMN filopodia; consistent with a role for Vangl2 and Fzd3a in the microenvironment, we show planar polarized localization of these proteins in the adjacent floorplate. Together, our findings support a model in which canonical interactions between PCP components within FBMNs and between the FBMNs and their planar polarized neuroepithelial environment promotes migration via the selective stabilization of FBMN filopodia.

## Results

### PCP signaling within FBMNs is required for their migration

**Fig 1:**
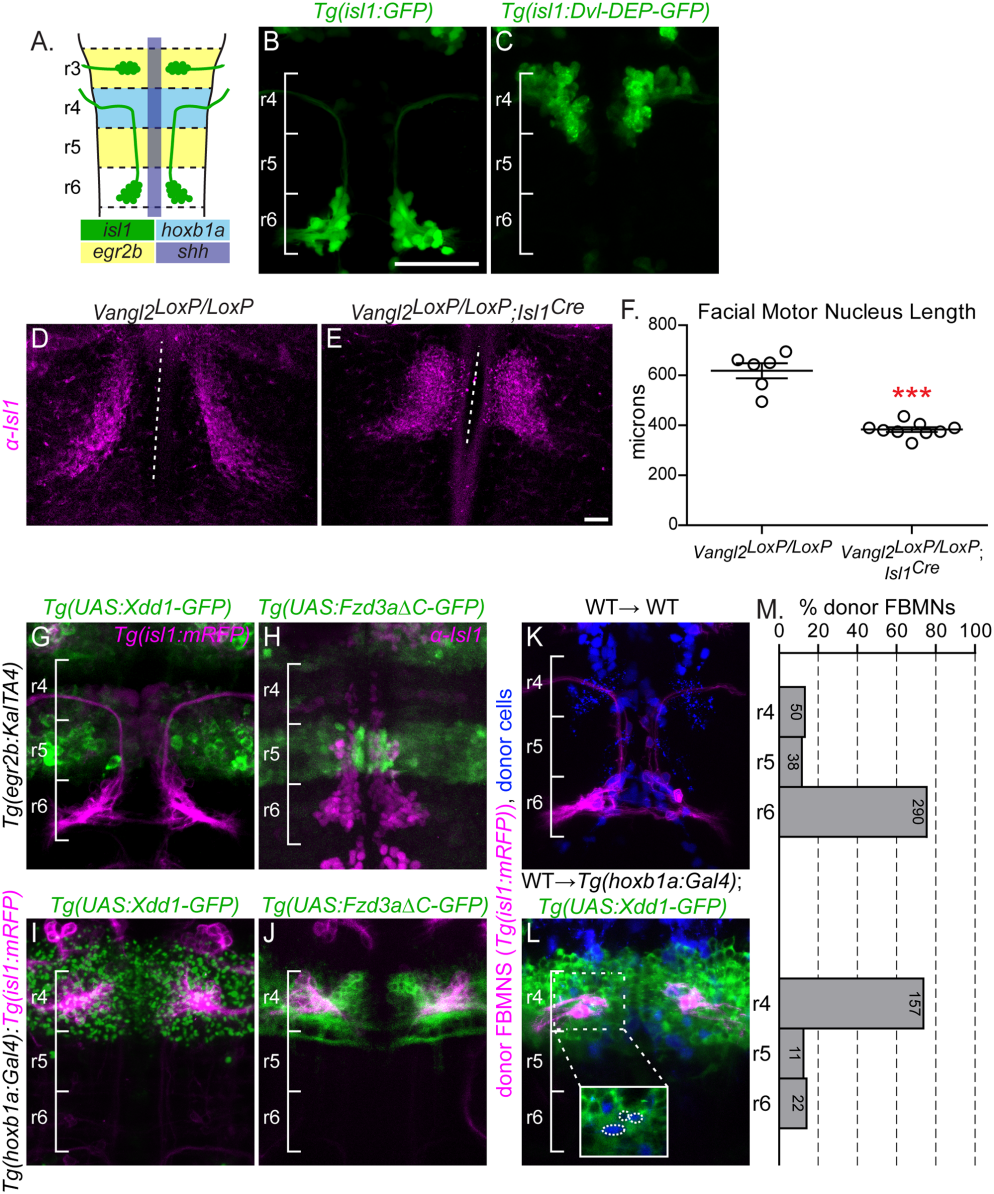
PCP signaling is required within FBMNs and in their r4 environment. (A) Schematic showing a dorsal view of a 48 hours post fertilization (hpf) zebrafish hindbrain with anterior to the top. Facial Branchiomotor neurons (FBMNs) (green) migrate posteriorly from rhombomere (r) 4 to r6, leaving a trailing axon that exits from r4. The enhancer element *islet-1* (*isl1*) CREST drives expression in branchiomotor neurons (green); the *hoxb1a* element drives expression in r4 (light blue); *egr2b* drives expression in r3 and r5 (yellow) and *shh* drives expression in the floorplate (purple). (B,C, G-L) Live or (H) fixed confocal images showing dorsal views of the hindbrain of 48 hpf zebrafish embryos with anterior to the top. Brackets mark rhombomere (r) position. Scale Bar: 50μm (B) *Tg(isl1*:*GFP)* expression in a wild type embryo at 48 hpf. (C) *Tg(isl1*:*Dvl-DEP-GFP)* embryo with unmigrated Dvl-DEP-GFP-expressing FBMNs in r4. (D,E) Dorsal view of E12.5 mouse hindbrains with FBMNs (magenta) labeled with anti-Isl1 antibody. Dotted lines indicate the length of facial motor nucleus. Scale Bar: 100μm (D) Migrating FBMNs in *Vangl2*^*LoxP/LoxP*^ control embryos. N = 6 embryos. (E) Blocked FBMNs in *Vangl2*^*LoxP/LoxP*^;*Isl1*^*Cre*^ embryos. N = 9 embryos. (F) Quantitation of FBMN migration stream length in *Vangl2*^*LoxP/LoxP*^ control embryos and *Vangl2*^*LoxP/LoxP*^;*Isl1*^*Cre*^ embryos. ***p = 0.0003. Significance was determined using an unpaired, two-tail t-test. (G-L) FBMNs (magenta) are either expressing *Tg(isl1*:*mRFP)*(G,I-L) or are stained with anti-Isl1 (H). (G,H) *Tg(egr2b*:*KalTA4)*-driven expression of *Tg(UAS*:*Xdd1-GFP)* (G) and *Tg(UAS*:*Fzd3aΔC-GFP)* (H), throughout r3 and r5 does not block FBMN migration. (I,J) *Tg(hoxb1a*:*Gal4)*-driven expression of *Tg(UAS*:*Xdd1-GFP)* (I) and *Tg(UAS*:*Fzd3aΔC-GFP)* (J), throughout r4 blocks FBMN migration out of r4. (K,L) Chimeric embryos with transplant conditions indicated as donor→ host. Cascade blue-dextran marks all donor-derived cells (blue) and *Tg(isl-1*:*mRFP)* marks all donor-derived FBMNs (magenta). (K) Wild type donor-derived FBMNs migrate normally in a non-transgenic control host. N = 37 embryos, 378 FBMNs. (L) Wild type donor-derived FBMNs fail to migrate out of r4 that is expressing *Tg(UAS*:*Xdd1-GFP)*. N = 26 embryos, 190 FBMNs. Inset: same image without the magenta channel showing that donor-derived FBMNs (blue, circled) are not themselves expressing Xdd1-GFP (green). (M) Histograms indicate the percent of donor-derived FBMNs at 48 hpf that failed to migrate (r4), migrated partially (r5) or migrated fully (r6). Each histogram corresponds to the chimeric condition in the image to its left and numbers indicate the number of FBMNs represented in each bar.

Initial chimeric analyses suggested that the PCP components Vangl2, Fzd3a, Celsr2 and Scrib primarily act non-cell-autonomously to regulate FBMN migration [31, 48, 49]. An additional cell-*autonomous* role for Vangl2 and Scrib in FBMN migration has been demonstrated [51], but refuted by others [55]. To determine whether PCP signaling is required cell-autonomously within FBMNs for their migration, we expressed a dominant negative (DN) form of the PCP core component Dvl specifically in branchiomotor neurons using the *islet-1* (*isl1*) CREST enhancer (Fig 1A, 1B and 1C) [56]. Dvl is the branching point between multiple Wnt signaling pathways, and the overexpression of its individual domains exert pathway-specific DN properties [57]. Work in multiple vertebrate systems has demonstrated that Xdd1 and Dvl-DEP, two truncated forms of Dvl, act as PCP-specific DNs [25–27].

In previous studies mRNA injection of these DNs failed to disrupt zebrafish FBMN migration [31, 54]. We reasoned that this could be due to decreased DN mRNA levels or activity by the time of FBMN migration. To stably express DN forms of Dvl in FBMNs we raised stable *Tg(isl1*:*Dvl-DEP-GFP)* zebrafish in which FBMNs express Dvl-DEP-GFP. In wild type embryos, FBMNs fully migrate to r6 by 48 hours post fertilization (Fig 1B). However Dvl-DEP-GFP expressing FBMNs largely fail to migrate, with 31/35 of *Tg(isl1*:*Dvl-DEP-GFP)* embryos displaying FBMN migration defects where most FBMNs (>75%) remain in in r4 (Fig 1C). This demonstrates that PCP signaling within FBMNs is required for their migration.

To further confirm this, and to test specifically whether the core transmembrane PCP component Fzd3a, like Vangl2 [51], is required within FBMNs for migration, we used chimeric analysis to assess the ability of *fzd3a*^*rw689*^ mutant FBMNs to migrate in a normal planar polarized neuroepithelium. In these experiments we prevented host FBMN migration using a *pk1b* morpholino since it is well known that migrating FBMNs can carry other FBMNs with them independent of PCP signaling, complicating the interpretation of chimeras [51, 58]. *pk1b* morphants precisely phenocopy *pk1b* mutants in which FBMNs fail to migrate even though the surrounding neuroepithelium can support wild type FBMN migration [50, 51, 59]. While 70.9% of wild type FBMNs migrate out of r4 in a *pk1b* morphant environment, only 19% of *fzd3a* mutant FBMNs do so (S1 Fig). This suggests that Fzd3a is required within FBMNs for migration.

The requirement for the core PCP components Vangl2, Fzd3 and Celsr1-3 is conserved in mouse FBMN migration [52, 54, 60]. In order to confirm a FBMN-autonomous requirement for PCP signaling we employed tissue-specific knockout of *Vangl2* in mouse FBMNs using a floxed *Vangl2* allele and *Isl1*-driven Cre recombinase [61, 62]. In the mouse embryo, FBMN migration occurs between E10.5 and E14.5 with neurons reaching r6 by E12.5 [45]. In E12.5 homozygous floxed animals (*Vangl2*^*LoxP/LoxP*^) lacking *Isl1*^*Cre*^ FBMNs migrate to r6 and the mean length of the migration stream is 618μm (Fig 1D and 1F; N = 6). In contrast FBMNs in *Vangl2*^*LoxP/LoxP*^; *Isl1*^*Cre*^ animals are significantly blocked in r4 with FBMNs occupying an average of 383μm along the hindbrain (Fig 1E and 1F; N = 9; p = 0.0003).

Taken together, the disruption of migration due to FBMN-restricted DN expression, our chimeric analysis of *fzd3a*^*-/-*^ and previous chimeric analysis of *vangl2*^*-/-*^ FBMNs [51] and the failure of FBMN migration after FBMN-specific disruption of *Vangl2* in the mouse confirms a FBMN-autonomous requirement for PCP signaling in migration.

### FBMN migration requires PCP signaling non-autonomously in r4

While these data support a cell-autonomous requirement for PCP signaling in FBMN migration, PCP signaling in FBMNs is not *sufficient* for their migration. Indeed, a non-autonomous requirement for PCP signaling in FBMN migration has been well established in chimeras in which wild type FBMNs are unable to migrate in *vangl2*, *fzd3a*, *celsr2* or *scrib* mutant hosts [31, 48, 49, 51]. Since PCP is a cell-contact mediated signaling pathway in which the same transmembrane protein components are required in both contacting cells [2], an attractive hypothesis is that FBMNs receive PCP cues from cells in their environment that promote or direct their migration. Thus we sought to determine where PCP signaling is required in the FBMN migratory path for migration.

To block PCP signaling in distinct compartments of the hindbrain, we used the Gal4/UAS system to drive rhombomere-restricted expression of *Tg(UAS*:*Xdd1-GFP)* as well as a C-terminally truncated Fzd3a *Tg(UAS*:*Fzd3aΔC-GFP)* that lacks its cytoplasmic region, which has been shown to function as a potent PCP DN tool in zebrafish [49]. We used *Tg(egr2b*:*KalTA4)* to drive expression throughout r3 and r5 starting at 12 hpf [63] and *Tg(hoxb1a*:*Gal4)* [64] to drive expression throughout r4 starting at 10 hpf (Fig 1A). Expression of Xdd1-GFP or Fzd3aΔC-GFP along the migration path in the r5 neuroepithelium does not affect migration (Fig 1G and 1H). In contrast, r4-restricted expression of Xdd1-GFP or FzdΔC-GFP completely blocks FBMN migration (Fig 1I and 1J). This suggests that PCP signaling is required at the onset, but not throughout the course of FBMN migration. However we note that r5 expression of Xdd1-GFP or Fzd3aΔC-GFP using *egr2b*:*KalTA4* comes on slightly later than r4 expression using *hoxb1a*:*Gal4* (12 hpf compared to 10 hpf), so the caveat remains that PCP signaling is not fully disrupted in r5 at the time of migration with the available tools.

It was not surprising that FBMNs fail to migrate in *Tg(hoxb1a*:*Gal4)*; *Tg(UAS*:*DN-GFP)* embryos given that FBMNs arise in r4, and thus express *hoxb1a* throughout their early development, and we had already shown a cell-autonomous requirement for PCP signaling within FBMNs. To assess whether PCP signaling plays a role in the r4 neuroepithelium outside of FBMNs, we transplanted wild type *Tg(isl1*:*mRFP)* donor FBMNs into the presumptive ventral hindbrain of *Tg(hoxb1a*:*Gal4)*; *Tg(UAS*:*Xdd1-GFP)* embryos and assessed the positions of donor-derived FBMNs at 48 hpf. In control hosts, 87% (328/378) of wild type donor-derived FBMNs migrated out of r4. In contrast, in hosts expressing Xdd1-GFP in r4, only 17% (33/190) of donor-derived wild type FBMNs migrate out of r4 (Fig 1K, 1L and 1M). Thus, expression of Xdd1-GFP throughout r4 significantly hinders wild type FBMNs from initiating migration (p<0.0001, χ^2^ = 207.8) (Fig 1M). This demonstrates that there is a non-autonomous requirement for PCP signaling for FBMN migration in r4.

### Environmental PCP signaling is required for the migration of post-mitotic FBMNs

Having established that PCP signaling is required both within FBMNs and their r4 neuroepithelial environment for migration to occur; we asked *when* this signaling is required. PCP signaling polarizes neuroepithelial progenitors before FBMNs differentiate [7, 65, 66]. It is possible that this early neuroepithelial polarity is maintained in FBMNs to orient their initial migration. Alternatively, PCP signaling active at the time of migration initiation may promote migration. We reasoned that in the former case a planar polarized environment would not be required for migration after FBMNs had differentiated while in the latter case PCP function in the r4 environment would continue to be essential for migration.

**Fig 2:**
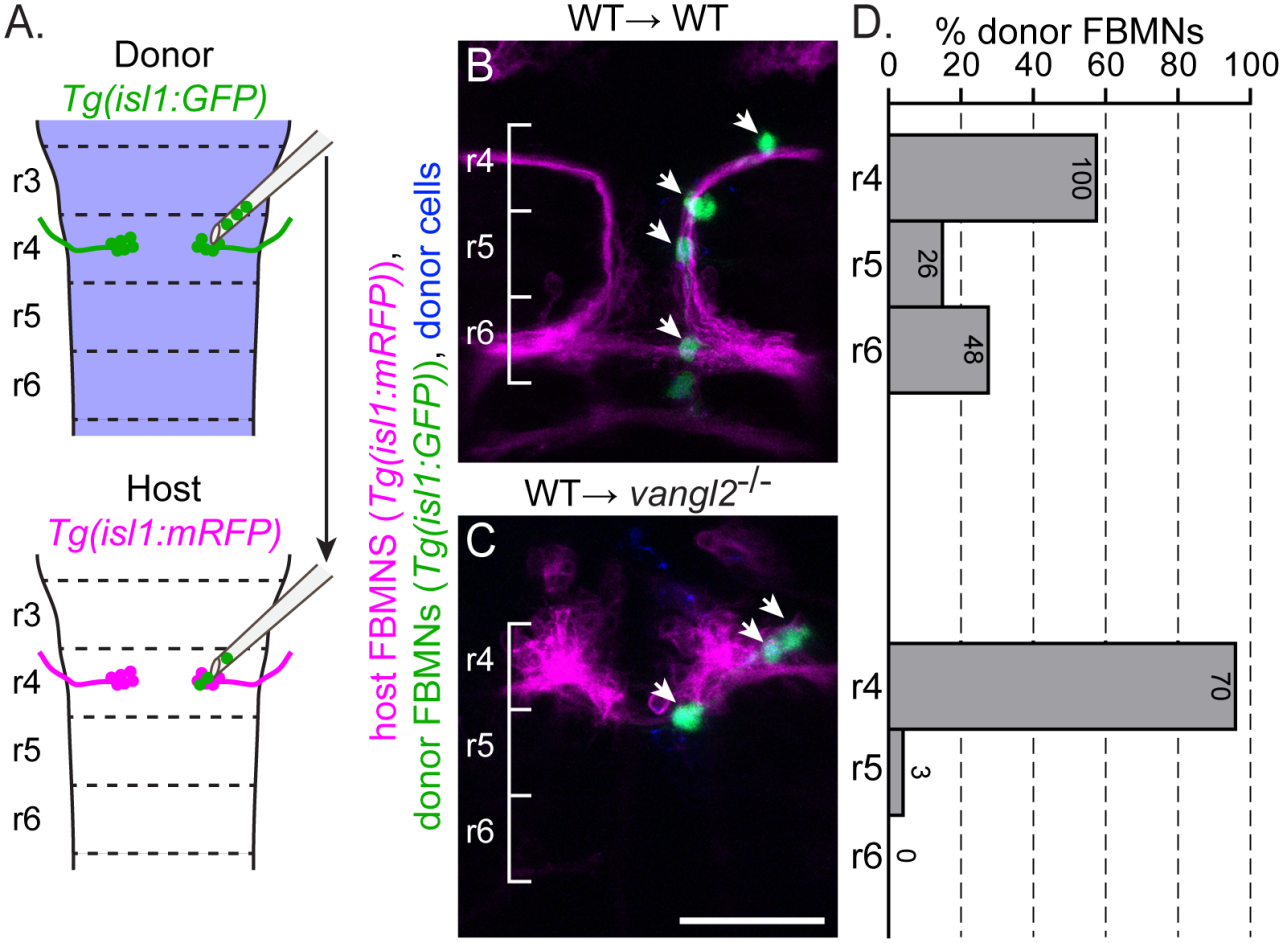
Post-mitotic FBMNs require PCP signaling for migration. (A) Schematic of the late stage FBMN transplantation procedure in which a small number (1–5) of post-mitotic, pre-migratory FBMNs are moved from r4 of a *Tg(isl1*:*GFP)* donor into r4 of a stage-matched, 16 hpf *Tg(isl1*:*mRFP)* host. (B, C) Live confocal images showing dorsal views of chimeras at 48 hpf with anterior to the top. Transplant conditions are indicated as donor→host. Cascade blue-dextran marks all donor-derived cells (blue), *Tg(isl1*:*mRFP)* marks host FBMNs (magenta) and *Tg(isl1*:*GFP)* marks donor-derived FBMNs (green). White arrows indicate donor-derived FBMNs at 48 hpf. (D) Quantitation of the percent of donor-derived FBMNs at 48 hpf that failed to migrate (r4), partially migrated (r5) or fully migrated (r6). Each histogram refers to the transplant condition in the image to its left and numbers indicate the number of FBMNs represented in each bar. WT→WT, N = 42 embryos, 174 FBMNs; WT→ *vangl2*^*-/-*^, N = 16 embryos, 73 FBMNs. ***p<0.0001 compared to WT→WT control. Brackets indicate rhombomere location. Scale bar: 50μm.

To determine when PCP signaling is required for FBMN migration, we transplanted a small number (1–5) of pre-migratory but post-mitotic FBMNs directly from r4 of a *Tg(isl1*:*GFP)* donor into r4 of a stage-matched wild type or *vangl2*^*m209*^ mutant *Tg(isl1*:*mRFP)* host (Fig 2A). During this extraction procedure, transplanted cells round up and become separated in the transplant pipette and are unlikely to retain polarity information. Nevertheless, 28% (48/174) of surviving post-mitotic FBMNs transplanted into a wild type host r4 migrated to r6 (Fig 2B and 2D). To rule out the possibility that the transplanted FBMNs are simply being carried by migrating host FBMNs, we transplanted post-mitotic FBMNs into a *pk1b* mutant host, which has normal neuroepithelial PCP but no host FBMN migration; in this environment 11%, (17/152) of transplanted FBMNs migrated (S2 Fig). This suggests that host neurons do contribute to migration [51, 58], but that post-mitotic transplanted FBMNs can migrate without contribution from migrating host neurons. Importantly, 0% (0/73) of FBMNs migrated to r6 after being transplanted into a *vangl2* mutant host (Fig 2C and 2D). Together, these results suggest that post-mitotic FBMNs engage PCP signaling as they initiate their migration out of r4.

### Floorplate PCP is not required for FBMN migration

**Fig 3:**
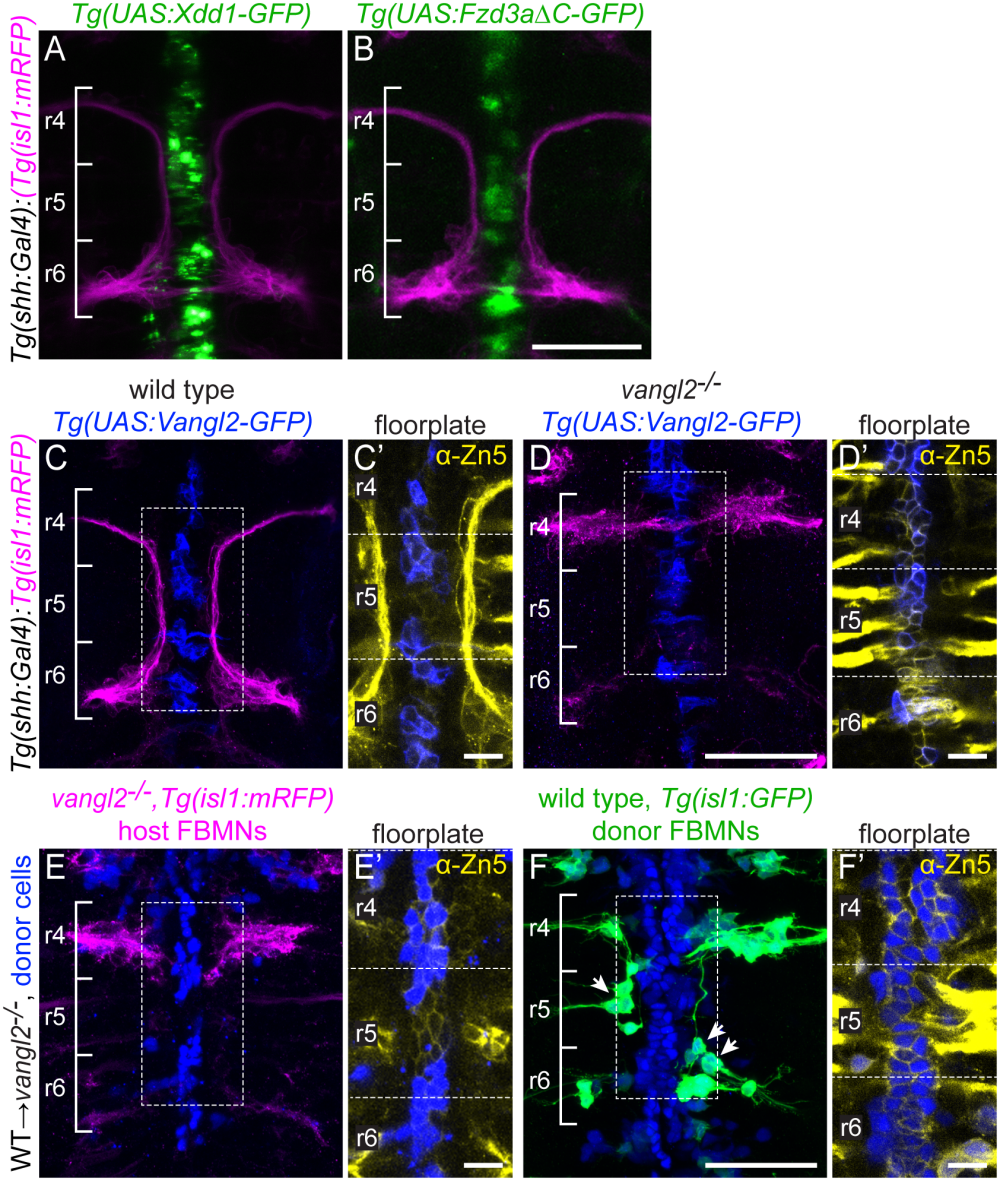
Floorplate PCP is neither required nor sufficient for FBMN migration but can support the migration of WT FBMNs. Confocal images showing dorsal views of 48 hpf hindbrains with anterior to the top. (A,B) *Tg(shh*:*Gal4)*-driven expression of *Tg(UAS*:*Xdd1-GFP)* (A) and *Tg(UAS*:*Fzd3aΔC-GFP)* (B) does not disrupt FBMN (magenta) migration. N = 13 Xdd1-GFP expressing embryos and 26 Fzd3aΔC-GFP expressing embryos. (C,D) *Tg(shh*:*Gal4)*-driven floorplate expression of GFP-Vangl2 (blue) in the floorplate of a wild type sibling does not disrupt FBMN migration (magenta) (C) and does not rescue migration in a *vangl2* mutant (D). N = 24 *vangl2* mutants with GFP-Vangl2 expression in the r4 floorplate, 14 with 5 or more expressing floorplate cells in r4. (C’,D’) Boxed regions from panels C and D respectively, showing a single z-plane where GFP-Vangl2 (blue) is expressed broadly in floorplate cells whose membranes are marked with the Zn5 antibody (yellow) [69]. (E,F) Genetic chimeras. Cascade blue-dextran marks all donor-derived cells (blue), *Tg(isl1*:*mRFP)* marks host FBMNs (magenta in E) and *Tg(isl1*:*GFP)* marks wild type donor-derived FBMNs (green in F). (E) The presence of wild type floorplate cells (blue) in a *vangl2* mutant host embryo does not rescue the migration of host FBMNs. N = 16 embryos with extensive contribution of WT cells to the floorplate. (F) The presence of wild type floorplate cells (blue) in a *vangl2* mutant can, however, support the migration of co-transplanted wild type donor derived FBMNs (green, arrows). N = 8 embryos with migrated donor-derived FBMNs/22 embryos with donor-derived FBMNs; N = 76 migrated FBMNs/383 total donor-derived FBMNs. (E’,F’) Single Z-planes of the boxed regions from panels E and F respectively, show that donor-derived cells (blue) are in the Zn-5-positive floorplate (yellow). Scale bars: 50 μm, 5μm in the insets.

FBMNs migrate in the ventral neural tube adjacent to the floorplate (Fig 1A, [49, 67]) making the floorplate a potential source of PCP signaling for FBMN migration. A recent report found that floorplate expression of Vangl2 is both necessary and sufficient for FBMN migration [55]. Here, to investigate whether PCP signaling in the floorplate is required for FBMN migration, we generated a *Tg(shh*:*Gal4)* line (see Methods) to drive Xdd1-GFP or Fzd3aΔC-GFP expression in the notochord and floorplate (S3A, S3B and S3C Fig). In order to determine if dominant negative expression does indeed disrupt floorplate planar polarity, we quantified the anterior-posterior position of the basal body in single floorplate cells as the ratio of its distance from the anterior membrane to the full anterior-posterior cell length (S3G Fig). Basal bodies in wild type floorplate cells are planar polarized to the posterior membrane (average positon = 78% of cell length, S3D and S3H Fig, [6]). Conversely, basal body planar polarization is significantly disrupted in floorplate cells expressing Xdd1-GFP or Fzd3aΔC-GFP (average position = 63% and 59% of cell length respectively; S3E, S3F and S3H Fig). By comparison, floorplate cells in *vangl2* mutants display a complete loss of basal body planar polarity (average position = 47% of cell length, [51]) (S3H Fig). With the caveat that this effect on floorplate planar polarity was scored after FBMN migration was complete (48 hpf) rather than at the onset of migration (18–24 hpf), DN expression in the floorplate in the floorplate had no effect on FBMN migration (Fig 3A and 3B). To confirm this, we specifically knocked Vangl2 out in the mouse floorplate using the floxed *Vangl2* allele described above [61] and *Shh-*driven Cre recombinase [68]. We found that Cre-mediated deletion of Vangl2 in the mouse floorplate does not disrupt FBMN migration (S4 Fig). These results suggest that PCP signaling in the floorplate is not required for FBMN migration, and point to the possibility that loss of PCP in the floorplate can be compensated for by other planar polarized cells in the r4 neuroepithelial environment.

Floorplate PCP could nevertheless be sufficient to rescue FBMN migration as has been suggested [55]. We tested the sufficiency of Vangl2 in the floorplate for FBMN migration in two ways. We expressed a GFP-Vangl2 fusion protein specifically in the floorplate of *vangl2* mutants and wild type siblings using stable *Tg(shh*:*Gal4)* driver and *Tg(UAS*:*GFP-Vangl2)* transgenic lines (*vangl2*^*m209/m209*^; *Tg(shh*:*Gal4); Tg(UAS*:*GFP-Vangl2)*). Although GFP-Vangl2 was expressed broadly in the floorplate in these otherwise mutant embryos starting at 14 hpf, and exhibits planar-polarized localization (S3A Fig and see below), it neither disrupted FBMN migration in a wild type embryo nor rescued migration in a *vangl2* mutant embryo (Fig 3C and 3D). Since a caveat of this experiment is that Vangl2 over-expression can itself disrupt planar polarity, we used targeted transplantation of wild type cells into the floorplate of *vangl2* mutants to test whether floorplate Vangl2 is sufficient to rescue FBMN migration. We never observed rescue of host FBMN migration in *vangl2* mutant *Tg(isl1*:*mRFP)* hosts with wild type donor-derived cells in the hindbrain floorplate (Fig 3E). This includes 9 cases with 10 or more wild type floorplate cells in rhombomere 4. This is contrary to the findings of Sittarmane et al. (2013) [55] who found that a single wild type floorplate cell in r4 of a *vangl2* mutant could rescue FBMN migration. Together, our findings suggest that Vangl2 function in the floorplate is not sufficient for FBMN migration.

In these transplant experiments we noted that FBMNs as well as floorplate cells differentiate from donor-derived cells. This is not unexpected, given the close proximity of floorplate and branchiomotor neuron progenitors in the early embryo [70]. Interestingly, we observed that unlike the mutant host FBMNs, wild type donor-derived FBMNs sometimes migrate (Fig 3F), and their ability to do so correlates with the number of wild type cells in the hindbrain floor plate (R^2^ = 0.244; p = 0.005). We conclude that Vangl2 function in the floorplate is not sufficient for FBMN migration, but that Vangl2 function in the floor plate can support the migration of *vangl2*^*+*^ FBMNs in an otherwise *vangl2* mutant neuroepithelium. Taken together, we conclude that the floorplate can serve as a source of PCP signals for FBMN migration, but other cells in the r4 environment, which are also planar polarized (see below) can compensate for the loss of normal floorplate PCP signaling.

### Vangl2 localization in the migratory environment

**Fig 4:**
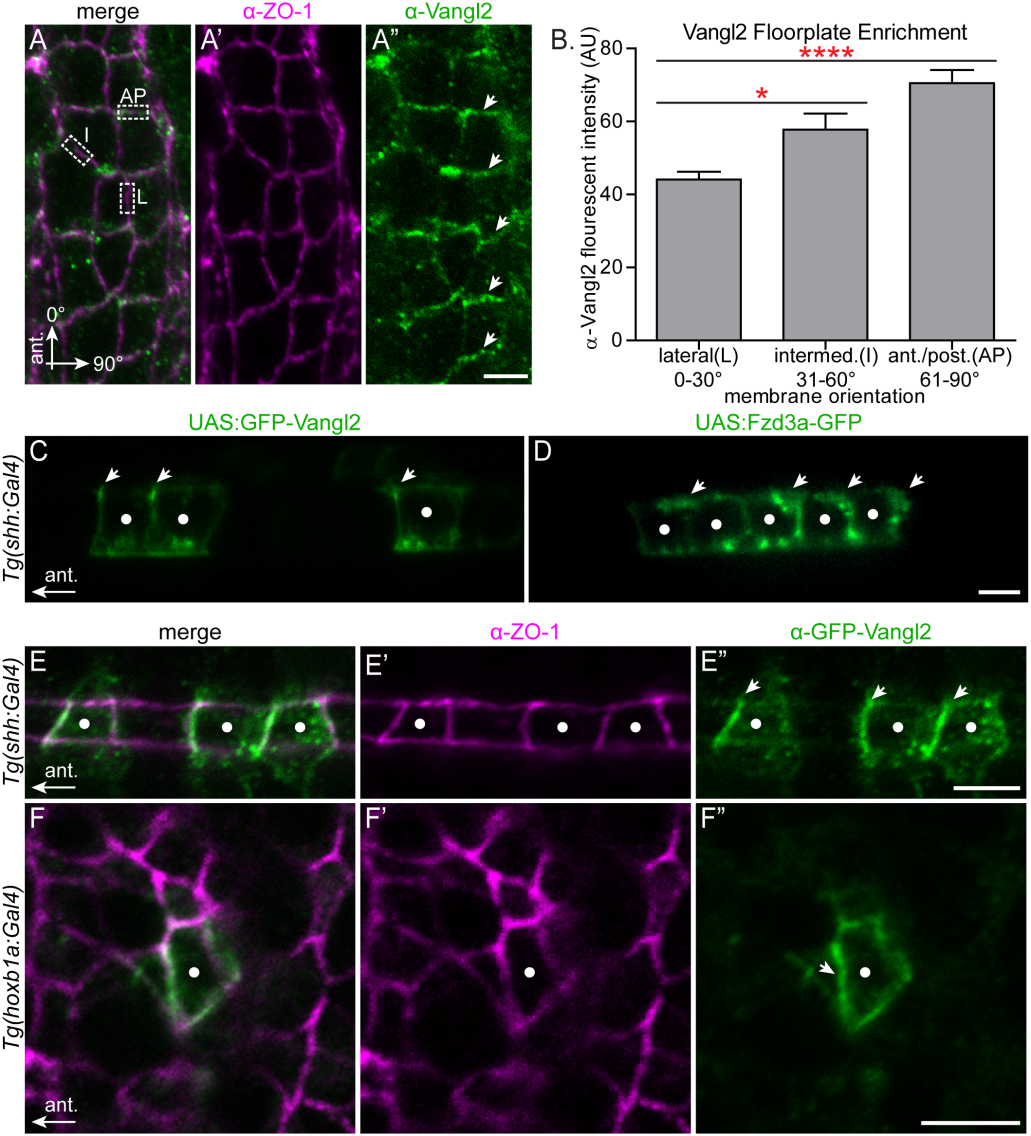
Polarization of PCP protein localization in the migratory environment. (A-A”) Dorsal view with anterior to the top of a 24 hpf wild type floorplate at the level of r4 co-immunostained with anti-Vangl2 (green) and anti-ZO-1 (magenta), a marker of apical tight junctions. The boxed regions in A are examples of anterior-posterior membranes (AP) (61–90° from AP axis), intermediate membranes (I) (31–60° from AP axis) and lateral membranes (L) (0–30° from AP axis). Arrows in A” indicate enrichment of anti-Vangl2 labeling at AP membranes. (B) Quantitation of fluorescent intensity of anti-Vangl2 labeling for AP, I and L membranes. N = 5 embryos, 192 membranes (57 L, 47 I, 88, AP). Graph represents data as mean ± SEM. *p = 0.018, ****p<0.0001; Significance was determined using a paired two-tail t-test with Welch’s correction. (C-D) Live lateral views of 48 hpf wild type floorplate cells at the level of the spinal cord with mosaic expression of GFP-Vangl2 (C) and Fzd3a-GFP (D). Anterior is to the left and dorsal/apical is up; white dots indicate the center of each expressing floorplate cell, arrows indicate anterior subapical membrane enrichment of GFP-Vangl2 (C) and posterior subapical enrichment of Fzd3a-GFP (D). (E-E”) Dorsal view of the apical surface of floorplate cells in a 48 hpf embryo expressing GFP-Vangl2 (green) and stained for ZO-1 (magenta) Anterior is to the left; white dots indicate the center of the expressing cell. Arrows in E” indicate anterior enrichment of GFP-Vangl2. (F-F”) *En face* view of the apical endfeet of neuroepitheilial cells in r4 of a 24 hpf embryo expressing GFP-Vangl2 (green) and stained for ZO-1 (magenta). Anterior is to the left; white dot indicates the center of the expressing cell. Arrow in F” indicates anterior enrichment of GFP-Vangl2. N = 17 embryos, 23 cells. Scale bars: 5 μm.

Thus far, we have shown that PCP signaling in FBMNs and their immediate neuroepithelial/floorplate r4 environment can drive migration. The localization of core PCP components is known to be crucial for many PCP mediated processes [2, 22]. Therefore, to better understand how PCP signaling might be used in neuronal migration we asked where PCP proteins localize within FBMNs and in their neuroepithelial microenvironment. Using a polyclonal antibody against zebrafish Vangl2, we observed localization of Vangl2 to cell membranes throughout the hindbrain neuroepithelium (S5 Fig). In the r4 floorplate, we noted a 1.6-fold enrichment of Vangl2 protein at anterior/posterior membranes of floorplate cells compared to their lateral membranes (Fig 4A and 4B). Co-staining with ZO1 shows that this staining is sub-apical, at the level of the tight junctions (Fig 4A’). In order to distinguish anterior from posterior membrane localization we mosaically expressed GFP-Vangl2 in the floorplate so we could visualize Vangl2 localization in isolated floorplate cells. This revealed that Vangl2 is specifically enriched at the anterior subapical membrane (Fig 4C and 4E). The normalized mean fluorescent intensity ratio of GFP-Vangl2 at the anterior membrane versus the posterior membrane in expressing floorplate cells is 2.2 (std. deviation 0.9; N = 29 cells in 8 embryos). Conversely, Fzd3a-GFP is enriched at the posterior membrane (Fig 4D). These findings for PCP protein localization in the floorplate are consistent both with the requirement for PCP core components in the posterior localization of the floor plate primary cilium [6], and with a conserved deployment of PCP core components in vertebrate and invertebrate epithelia.

While the regular organization of floorplate cells makes it easy to visualize their planar polarization, our findings suggest that the primary source of environmental PCP signaling in FBMN migration comes from neuroepithelial progenitor cells outside of the floorplate. Previous studies demonstrated a planar polarization of GFP-Pk and GFP-Vangl2 in neuroepithelial progenitor cells during zebrafish neurulation [7, 65], and of endogenous Vangl2 in the Xenopus neural plate [66]. We asked whether neuroepithelial progenitor cells display planar polarization of Vangl2 in r4 at the time of FBMN migration. Using the *Tg(hoxb1a*:*Gal4)* line we mosaically expressed GFP-Vangl2 and observed a subtle but significant asymmetry of GFP-Vangl2 to the anterior sub-apical side of r4 neural progenitors. While GFP-Vangl2 polarization is subtle and not detectable in all expressing neuroepithelial progenitors, in blinded experiments we were able to correctly guess the A-P orientation of embryos based exclusively on GFP-Vangl2 localization in r4 progenitors in 18/23 mosaically expressing embryos (p = 0.004 that 18/23 correct guesses were due to chance alone). The normalized mean fluorescent intensity ratio of GFP-Vangl2 at the anterior membrane versus the posterior membrane in cells where asymmetry is detectable is 1.82 (std. deviation 0.47, N = 17 embryos, 23 cells.) (Fig 4F). Thus both the r4 neuroepithelium and floorplate exhibit planar polarized Vangl2 localization at the time of FBMN migration.

### Vangl2 is enriched at the tips of retracting FBMN filopodia

**Fig 5:**
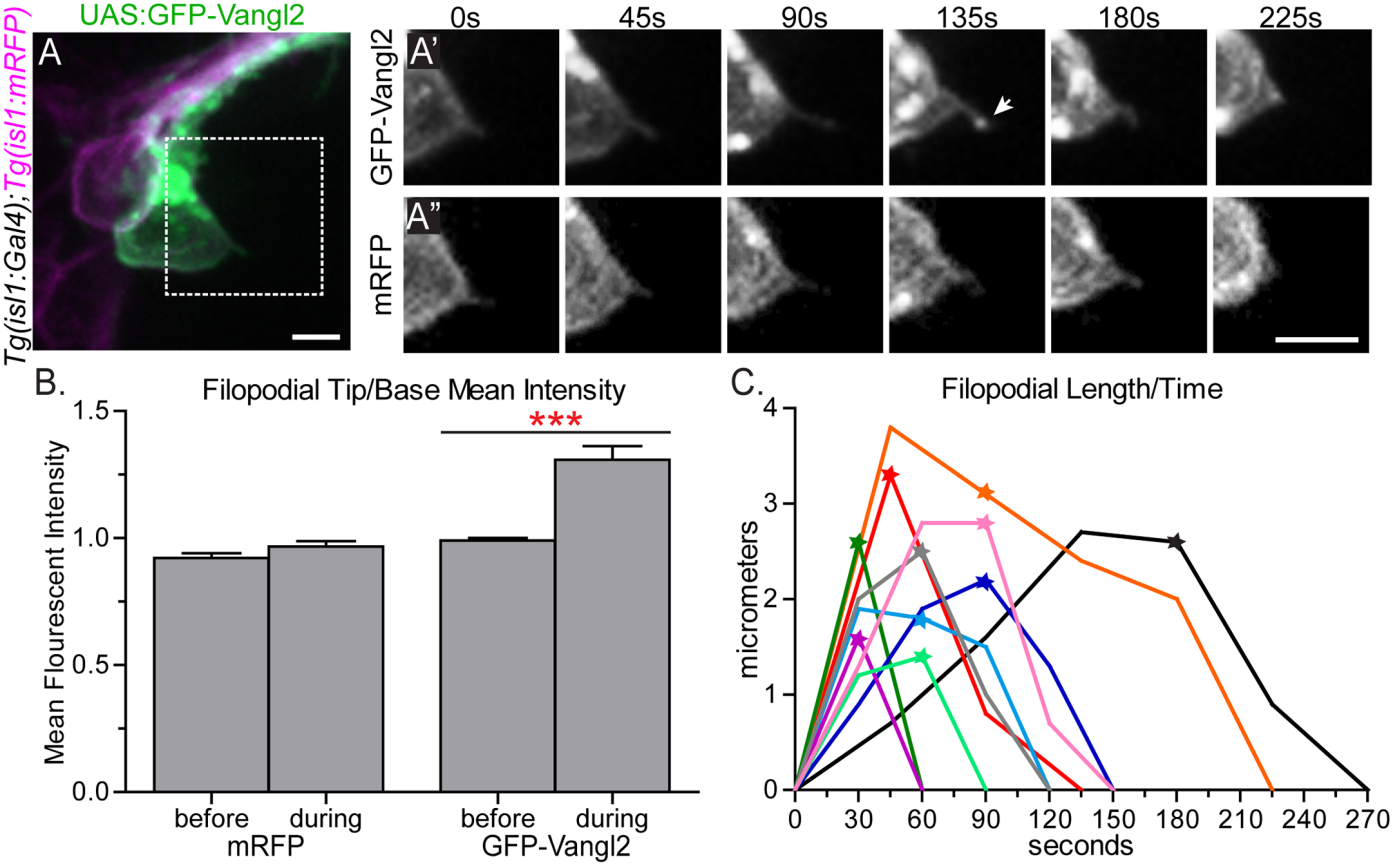
GFP-Vangl2 is enriched to the tip of retracting FBMN filopodia. (A) Live confocal image of a single GFP-Vangl2 expressing FBMN (green) in a *Tg(isl1*:*mRFP)* (magenta) 24 hpf embryo. Scale bar: 10 μm. (A’,A”) Magnified views of the boxed region in A of the individual channels, GFP-Vangl2 and *Tg(isl1*:*mRFP)* respectively, at the time points indicated. The arrow in E’ indicates enrichment of GFP-Vangl2 at the filopodial tip. Scale bar: 10 μm. (B) Quantitation of filopodia tip/base mean fluorescent intensity ratio for mRFP and GFP-Vangl2 at the time-point before and during GFP-Vangl2 enrichment. Before enrichment the mean fluorescent intensity ratio of GFP and mRFP at the filopodia tip versus the filopodia base is approximately 1 (N = 9 filopodia). During the enrichment event this ratio for GFP-Vangl2 is 1.31 while the ratio remains close to 1 for mRFP (N = 12 filopodia). (C) Plot showing the change in filopodial length over time for 10 filopodia. The stars indicate the time-point that GFP-Vangl2 is enriched at each filopodium tip. The black trace corresponds to the filopodium in A’,A”. Graph represents data as mean ± SEM. ***p<0.001; Significance was determined using an unpaired, two-tail t-test with Welch’s correction.

We next sought to determine where Vangl2 localizes in migrating FBMNs. Endogenous Vangl2 in FBMN membranes and the membranes of surrounding cells could not be resolved using the anti-Vangl2 antibody and, unlike static floorplate cells and neuroepithelial progenitors, FBMNS are highly dynamic, extending primarily filopodia-like protrusions as they migrate [51, 71]. Reasoning that Vangl2 localization would be similarly dynamic, we mosaically expressed GFP-Vangl2 in FBMNs and visualized localization using spinning disc time-lapse imaging. We found that GFP-Vangl2 localizes throughout the membrane as well as in putative cytoplasmic vesicles, as is predicted for a transmembrane protein (Fig 5A). However, in addition to its membrane localization, we observe transient enrichment of GFP-Vangl2 at the tips of a subset filopodia immediately preceding filopodia retraction (Fig 5A’–5C and S1 Movie). Before enrichment the mean fluorescent intensity ratio of GFP at the filopodia tip versus the filopodia base is approximately 1 (0.99 ± 0.01), as is the case for mRFP (background membrane marker) (0.92 ± 0.02). During the enrichment event, this ratio for GFP-Vangl2 increased to 1.31 ± 0.05 while the ratio for mRFP remained close to 1 (0.97 ± 0.02) (Fig 5B). Since the ratio for mRFP remained close to 1, this suggests that the enrichment of GFP-Vangl2 correlates with increased Vangl2 protein levels at filopodia tips and not simply condensation of the membrane due to retraction. Furthermore, as described below, addition of exogenous GFP-Vangl2 in FBMNs results in a reduced filopodial lifetime which is opposite to the effect of loss of Vangl2 in FBMNs. This suggests that exogenous GFP-Vangl2 is functioning in FBMNs and that this observed localization of Vangl2 at the tips of filopodia is correlated with retraction. This enrichment of GFP-Vangl2 in filopodia never lasted for more than one time-point (images were taken at 30–45 second intervals) and was only detected in a subset of filopodia (N = 11/84 filopodia on 8 neurons in 7 embryos); it is likely that due to the transient nature of enrichment events and the constraints of our imaging rate we failed to observe many enrichment events. Importantly, however, the enrichment events we captured invariably preceded filopodial retraction; filopodia never extended further after an enrichment event (Fig 5C). Consequently, we infer that Vangl2 may function in FBMN filopodia to signal retraction events.

### Vangl2 and Fzd3a function cell-autonomously to regulate FBMN filopodial activity in an antagonistic manner

Our findings that PCP signaling is required within FBMNs for migration, and that Vangl2 localizes transiently to the tips of retracting filopodia, suggested the possibility that PCP signaling influences filopodial dynamics in migrating neurons *in vivo*. In order to determine the cellular basis of FBMN migration defects in PCP mutants, we imaged the protrusive dynamics of single mutant FBMNs at high resolution *in vivo*. Previous studies have described membrane protrusions in fixed or live embryos expressing cytoplasmic GFP or membrane-RFP in bulk FBMNs at low time resolution, however the overlap between FBMNs allows only a subset of protrusions to be visualized and their dynamics could only be inferred from distant time points [31, 55, 58, 71]. To visualize the protrusive activity of single FBMNs at high time resolution, we utilized cell transplantation to generate embryos in which one or a few FBMNs express membrane-localized teal fluorescent protein (*Tg(isl1*:*mTFP)*), and imaged protrusion dynamics of single FBMNs at 30-second intervals, the shortest interval at which we could acquire comprehensive z-stacks on our instruments. We focused on the function of Vangl2 and Fzd3a, the mutually antagonistic transmembrane core components, whose localized activity is both the hallmark and the driver of classical epithelial planar polarity [2].

**Fig 6:**
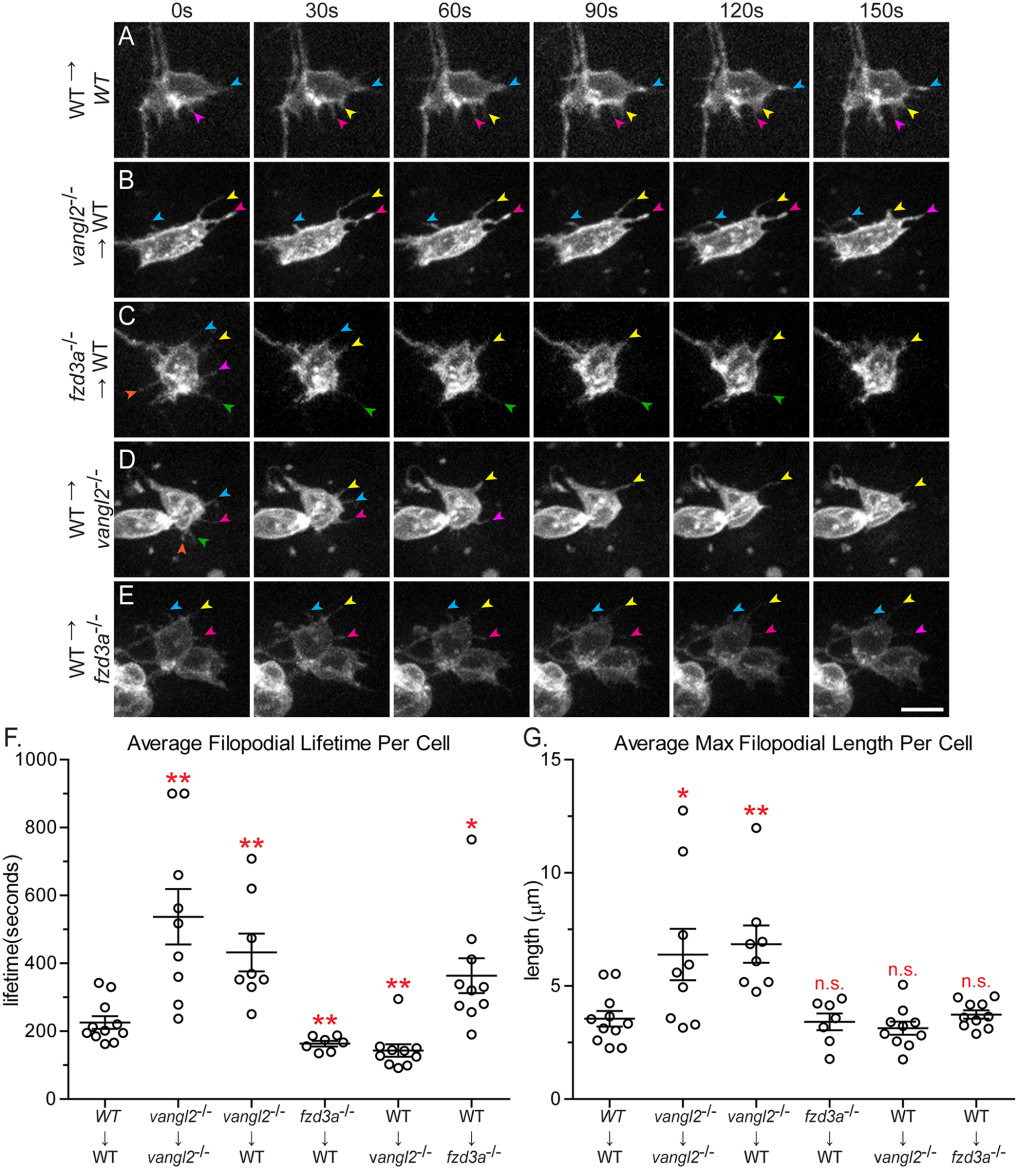
Vangl2 and Fzd3a have opposing cell-autonomous and non-cell-autonomous roles in modulating filopodial dynamics. (A-E) Time-lapse spinning-disc confocal series of donor-derived FBMNs in chimeric embryos at 24–30 hours post-fertilization (hpf). Transplant conditions are indicated on the left as donor→host. Colored arrows indicate individual filopodia at different time-points. Anterior is to the top and medial is to the right. Scale bar: 5 μm. (F) Quantitation of filopodial lifetime for donor-derived FBMNs. Each data point is an average of all the filopodial lifetimes for one FBMN. (G) Quantitation of the maximum filopodial length for donor-derived FBMNs. Each data point is the average maximum length for all the filopodia of one FBMN. WT→WT: N = 6 embryos, 11 neurons (3 in r4, 4 in r5, 4 in r6), 70 filopodia; *vangl2*^*-/-*^→*vangl2*^*-/-*^: N = 6 embryos, 9 neurons, 43 filopodia; *vangl2*^*-/-*^→WT: N = 6 embryos, 8 neurons, 44 filopodia; *fzd3a*^*-/-*^→WT: N = 7 embryos, 7 neurons, 73 filopodia; WT→ *vangl2*^*-/-*^: N = 8 embryos, 10 neurons, 152 filopodia; WT→ *fzd3a*^*-/-*^: N = 6 embryos, 10 neurons, 65 filopodia. Graphs represent data as mean ± SEM. *p<0.05, **p<0.01 compared to WT→WT control; n.s., not significant. Significance was determined using an unpaired, two-tail t-test with Welch’s correction.

Time-lapse imaging of FBMN membranes and f-actin dynamics revealed that filopodia are the prevalent protrusion type in FBMNs (Fig 6A–6E and S2 Movie). To determine whether PCP affects the polarized orientation of filopodia on migrating cells, we quantified the positions of filopodia on single isl1:mTFP-expressing FBMNs by measuring the angle from the anterior-posterior axis of a vector from the center of the cell to the base of each filopodium (S6A Fig). When wild type FBMNs are in r4, protrusive activity is largely radial 46.4% (13/28) of filopodia are located in the anterior half of the neuron, while 53.6% (15/28) of filopodia were on the posterior side (S6A and S6B Fig). Once neurons are migrating through r5 and r6, membrane protrusive activity becomes highly enriched posteriorly, in the direction of migration with 84.6% (44/52) of filopodia on the posterior side of the cell (S6C and S6D Fig). However, filopodia on *vangl*2 mutant FBMNs fail to polarize. Time-lapse images were collected at a developmental time-point at which wild type neurons would have already migrated out of r4. Similar to wild type neurons in r4, filopodia in *vangl2* mutant FBMNs are fairly evenly distributed along the anterior-posterior axis with 47.5% (29/61) of filopodia located anteriorly and 52.5% (32/61) located posteriorly (S6E and S6F Fig). These findings suggest that PCP signaling through Vangl2 is required to properly localize cytoskeletal dynamics in FBMNs.

To characterize protrusive membrane dynamics we quantified filopodial lifetime (number of seconds each filopodium is present during a 15 minute time-lapse period) and filopodial maximum length (the greatest length during the lifetime of filopodia lasting 90 seconds or longer) of *Tg(isl1*:*mTFP)* FBMNs. Wild type FBMNs generate filopodia with an average lifetime of 224.5 ± 18.66 (SEM) seconds and an average maximum length of 3.6 ± 0.3 μm (Fig 6A, 6F and 6G and S3 Movie). This filopodial lifetime is comparable to that observed in other vertebrate cells both *in vivo* and in culture [72–75]. When compared to wild type, FBMNs in *vangl2* mutant embryos have much more stable filopodia with a longer average lifetime of 537.3 ± 81.78 seconds (Fig 6F, S4 Movie; p = 0.0059). Filopodia of these *vangl2* mutant FBMNs also reach a greater average maximum length of 6.4 ± 1.1 μm (Fig 6G; p = 0.0406). We saw a similar trend when we used microinjection rather than transplantation to mosaically express mTFP in FBMNs in wild type and *vangl2* mutant embryos. These results suggest that Vangl2 is required to destabilize FBMN membrane protrusions. Previous studies have demonstrated that FBMNs in *vangl2* mutants move more slowly than wild type FBMNs and in random directions, which is consistent with Vangl2 being required to destabilize protrusions [31].

Since Vangl2 is required within FBMNs *and* their r4 microenvironment for migration, we sought to determine where Vangl2 functions to regulate filopodia dynamics. To determine the cell-autonomous function of Vangl2, we transplanted *vangl2* mutant FBMNs into a wild type host. Donor embryos carried the *Tg(isl1*:*mTFP)* transgene to visualize FBMNs and contained rhodamine dextran to track other donor-derived cells so we could ensure that donor-derived FBMNs were in fact in a genetically chimeric environment (S7 Fig). We found that *vangl2* mutant FBMNs in a wild type environment have longer, more stable filopodia with a mean lifetime of 432.0 ± 55.65 seconds and a maximum length of 6.8 ± 0.8 μm, similar to *vangl2* mutant FBMNs in a *vangl2* mutant host (Fig 6B, 6F and 6G and S5 Movie; p = 0.005 and p = 0.0078 respectively). To further test if Vangl2 functions cell-autonomously to control FBMN protrusive dynamics, we mosaically expressed GFP-Vangl2 in wild type FBMNs. FBMNs expressing GFP-Vangl2 in wild type embryos have less stable filopodia compared to wild type FBMNs, with an average lifetime of 123.4 ± 14.27 seconds (S1 Movie; N = 6 embryos, 7 neurons, 42 filopodia, p = 0.0013). Together, these loss- and gain-of-function findings suggest that Vangl2 functions within FBMNs to destabilize filopodia, since filopodia are affected in *vangl2* mutant and GFP-Vangl2-expressing FBMNs regardless of the genotype of cells in their microenvironment.

Fzd3a, like Vangl2, is required cell-autonomously and cell non-autonomously for FBMN migration (S1 Fig, [49]). To determine whether Fzd3a has a cell-autonomous role in FBMN protrusive activity, we transplanted *fzd3a* mutant FBMNs into a wild type host. We found that filopodia of *fzd3a* mutant FBMNs are significantly less stable than filopodia of wild type neurons, with a mean lifetime of 163.3 ± 8.006 seconds (Fig 6C and 6F and S6 movie; p = 0.0092). However, mean maximum filopodia length (3.4 ± 0.4 μm) was not significantly different than that of wild type FBMNs (Fig 6G). This suggests that Fzd3a normally functions within FBMNs to stabilize filopodia protrusions, consistent with a conserved role for Fzd in actin polymerization [11–13]. Taken together, our results suggest that Vangl2 and Fzd3a function antagonistically within FBMNs to regulate filopodial stability.

Given that we observed this cell-autonomous function for Vangl2 and Fzd3a in regulating FBMN membrane protrusions, we asked whether these proteins regulate FBMN protrusive dynamics independently of cells in the migratory environment. To address this question, we analyzed the protrusive dynamics of isolated FBMNs in primary culture. We found that cultured FBMNs display altered filopodial dynamics compared to FBMNs *in vivo*. Cultured wild type FBMNs have a mean lifetime of 537.5 ± 32.28 seconds (during a 600 second time-lapse) and an average maximum length of 6.0 ± 0.5 μm (S9 Fig). Furthermore, there is no difference in filopodial dynamics between cultured wild type and cultured *vangl2* mutant FBMNs (S9 Fig). This suggested to us that the cell-autonomous functions we observe for Vangl2 and Fzd3a *in vivo* depend on interactions with cells in the migratory environment.

### Vangl2 and Fzd3a have opposing cell *non-*autonomous functions in regulating FBMN filopodial activity

Since Fzd3a and Vangl2 are also required non-autonomously for FBMN migration and since FBMN protrusive dynamics depend on cells in the migratory environment, we asked whether cells in the FBMN environment influence FBMN protrusive activity in a PCP-dependent manner. In order to assess the role of Vangl2 in the environment, we imaged protrusion dynamics of wild type FBMNs in a *vangl2* mutant environment. Interestingly we found that wild type FBMNs have less stable filopodia in a *vangl2* mutant environment compared to a wild type environment, with a mean lifetime of 143.3 ± 18.28 seconds (Fig 6D and 6F and S7 Movie; p = 0.0056). The decrease in the average filopodia lifetime of wild type FBMNs in a *vangl2* mutant environment is largely due to these neurons having a larger proportion of filopodia present for only one (30 seconds) or two (60 seconds) time-points (S8 Fig). The mean average length however was not different between wild-type FBMNs in a wild type environment and wild type FBMNs in a *vangl2* mutant environment (Fig 6G) (3.1 ± 0.2 μm). Removing Fzd3a from the migratory environment had the opposite effect on FBMN filopodia. Wild type neurons in a *fzd3a* mutant environment generate dramatically more stable filopodia compared to those in a wild type environment, with a mean lifetime of 363.8 ± 51.12 seconds (Fig 6E and 6F and S8 Movie; p = 0.0273). Together our results suggest that the core PCP components Vangl2 and Fzd3a antagonize each other’s activity to control filopodial dynamics during neuronal migration *in vivo* and they do so by functioning both within FBMNs and in cells in their migratory environment.

## Discussion

**Fig 7:**
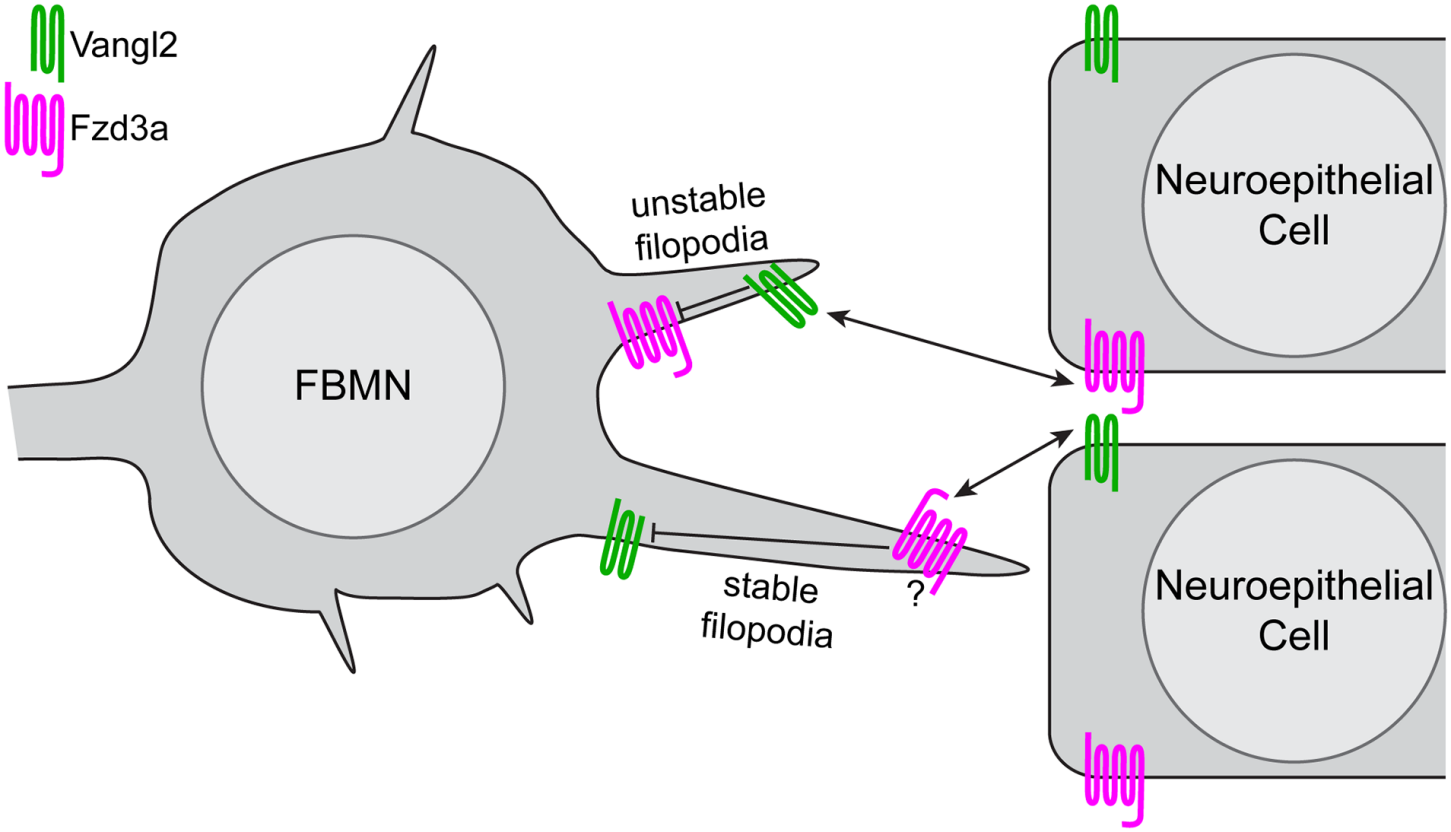
Model of PCP regulation of directed neuron migration. Based on the filopodial dynamics and migratory behaviors of FBMNs we observed in genetic chimeras, and the localization of Vangl2 and Fzd3a we observed in FBMNs and the cells of their migratory environment, we suggest a model in which antagonistic interactions between Vangl2 and Fzd3a mediate the observed effects on FBMN filopodial dynamics and through them, directional neuron migration. Within FBMNs, Vangl2 (green) localizes to filopodial tips and destabilizes them while Fzd3a (magenta) has the opposite, stabilizing effect. In the planar-polarized cells of the migratory environment Vangl2 serves to stabilize filopodia while Fzd3a destabilizes them. In light of the known intracellular and intercellular interactions between Vangl and Fzd that underlie epithelial planar polarization, we hypothesize that interactions between Fzd3a and Vangl2 complexes destabilize one another intracellularly while they promote one another’s effects on the actin cytoskeleton when they interact across cell membranes. Whether these interactions provide *directional* cues for migration remains to be discovered.

Based on our findings, we propose a model for the role of PCP signaling in FBMN migration in which canonical interactions between the transmembrane PCP core components Vangl2 and Fzd3a regulate filopodial dynamics, thereby signaling and/or regulating adhesion for directional migration (Fig 7). We note that this model for filopodial dynamics is based on genetics and that our work does not elucidate the molecular nature of these interactions, which remain controversial even in the context of epithelial polarity [17, 76]. This model is consistent with 1) a dual cell-autonomous and cell-non-autonomous requirement for PCP core components, specifically for the transmembrane components Vangl2 and Fzd3a, in FBMNs and their rhombomere 4 environment for migration; (this work, [31, 49, 51]); 2) the cytoskeletal and conserved molecular planar polarization of the r4 neuroepithelial environment including the floorplate (this work, [6]); 3) the ability of the planar polarized floorplate to promote the migration of wild type but not mutant FBMNs; 4) the localization of Vangl2 to retracting FBMN filopodial tips; 5) the antagonistic cell-autonomous roles of Fzd3a and Vangl2 in FBMN filopodial stability and 6) the opposite roles of Fzd3a and Vangl2 in the FBMN environment on FBMN filopodial stability.

### Mutual antagonism of Vangl2 and Fzd3a

Our *in vivo* observations of filopodial dynamics in genetic chimeras demonstrate an antagonistic intracellular relationship between Vangl2 and Fzd3a in migrating FBMNs that regulates the stability of filopodium-like protrusions. While occurring in the context of a highly dynamic structure, this antagonistic relationship of Vangl2 and Fzd3a is reminiscent of the situation in stably polarized epithelia, where mutual intracellular antagonism between Fzd and Vangl complexes sets up polarized actin dynamics within the cell, with Fzd activating actin polymerization distally and Vangl suppressing it proximally [11–13, 77, 78]. This conserved interaction between Fzd promoting and Vangl suppressing actin growth may be common to other migratory cells. In metastatic breast cancer cells induced by stromal Wnt11-containing exosomes, Fzd6 and Vangl1 exhibit mutually exclusive localizations, with Fzd6 on the leading edge of cell protrusions and Vangl1 on non-protrusive cell surfaces, and knock-down of either protein decreases cell motility [44]. Similarly in migrating leukemia cells, Dvl-3 (part of the Fzd complex) localizes to the leading edge while Vangl2 localizes to the trailing edge [79]. During mesodermal and neuroectodermal convergence, mediolaterally oriented cell surfaces exhibit increased actomyosin contractility [33, 80] that correlates with the asymmetric localization of PCP components Dvl and Pk (part of the Vangl complex) [7, 81], suggestive of a conserved intracellular antagonism of these complexes mediating actin dynamics. In contrast, in commissural growth cones, Vangl2 *promotes* Fzd3-dependent outgrowth induced by diffusible Wnt5a by antagonizing a non-canonical inhibitory interaction between Dvl1 and Fzd3 identified in that context [41]. These examples show that core PCP components localize to discrete domains of moving cells and we have shown *in vivo* for the first time that this results in opposing effects on filopodial stability.

### The role of filopodia in FBMN cell migration

Filopodia are commonly associated with the promotion of directed cell migration, although in some instances, axons and cells can achieve proper targeting and guidance without filopodia [82–84]. Due to their dynamics and long thin architecture, filopodia are capable of probing a wide area around cells, and they can contain receptors for diverse diffusible and membrane-bound signals and extracellular matrix molecules [85]. Thus, it is thought that the primary function of filopodia is as “antennae” that cells use to sense their microenvironment to orient directed cell migration [86]. Indeed it has been demonstrated that elimination of filopodia in axon growth cones does not impair axon outgrowth, but instead impairs growth cone turning in response to environmental cues [87–89]. This sensing role for filopodia has also been demonstrated in cell migration [84, 90]. In addition to a sensing role, filopodia are thought to contribute directly to cell motility, as cells lacking filopodia tend to migrate more slowly due to the absence of filopodial adhesion molecules which could induce traction and also through force generated by actin streaming in filopodia [82, 90–93]. In the context of FBMN migration, filopodia extend in all directions from neurons when they initiate their migration, and we see no bias in the orientation of the filopodia that are affected in PCP mutants. We hypothesize that filopodia act as sensors of asymmetrically localized cell-surface PCP components on the neuroepithelial cells through which they are migrating and that this sensing fine tunes filopodium dynamics such that these filopodia can promote migration by acting as force generators or appropriately sensing other, as-yet unidentified environmental cues. In other migrating cells, several effectors have been identified as possible links between PCP signaling and cytoskeletal regulation [33, 94, 95]. While our work does not elucidate how those signals are transduced to the filopodial actin cytoskeleton in FBMNs, our previous work identified the WAVE-homology domain containing actin regulator Nhsl1 as localizing to FBMN filopodia and being required cell-autonomously for FBMN migration [51, 96]; we hypothesize that PCP signals may be transduced to the actin cytoskeleton in FBMNs via Nhsl1.

### Fzd3a and Vangl2 function in the migratory environment

A more surprising finding than opposing cell autonomous roles for Fzd3a and Vangl2 in FBMN filopodial dynamics and migration is that the same PCP components function in the FBMN environment to influence filopodial dynamics but in the opposite way: Fzd3a in the environment destabilizes filopodia while Vangl2 in the environment stabilizes them. These non-autonomous functions for Fzd3a and Vangl2 in filopodial dynamics correlate with their non-autonomous roles in FBMN migration [31, 49]. Again, this is reminiscent of classical planar-polarity, where localized Fzd activity depends on the presence of Vangl in adjacent cells in the epithelium and vice versa; this is the mechanism by which PCP is coordinated across an epithelium [17–21]. We hypothesize that the cell-autonomous activities of Fzd3a and Vangl2 are activated in different filopodia when they contact Vangl2 and Fzd3a domains of neuroepithelial cells in the r4 environment (Fig 7), with consequences on the actin dynamics regulating filopodial stability, leading to changes in signaling and/or adhesion. We have shown here that Vangl2 and Fzd3a exhibit planar polarized localization in the r4 neuroepithelium and floorplate at the time of FBMN migration. In PCP mutants, this polarized information is absent and/or cannot be correctly interpreted by filopodia resulting in a failure of directional cell migration. We note that the cell-autonomous filopodial phenotypes appear to be dominant, since in constitutive mutants filopodia have the cell-autonomous phenotype (long and stable in *vangl2* mutants; unstable in *fzd3a* mutants). Together our findings suggest that conserved intracellular and intercellular interactions between PCP core components can have divergent effects on actin dynamics and consequently on cell behaviors.

While the similar effects on filopodial dynamics when Vangl2 is depleted from FBMNs and when Fzd3a is depleted from their environment suggest that the two proteins are working together, environmental PCP may also influence filopodia dynamics of FBMNs through an indirect mechanism. For instance, core PCP proteins have been implicated in the trafficking and regulation of membrane levels of cadherins in fly and in vertebrate epithelial cells [97–99]. Therefore, Vangl2 and Fzd3a in the migratory environment may modulate FBMN filopodia dynamics by regulating N-cadherin levels at the surface of neuroepithelial cells. Another potential mechanism by which PCP in the migratory environment may regulate FBMN filopodial dynamics is through regulation of membrane type-1 matrix metalloproteinase (MMP14), which are known to degrade extracellular matrix proteins. During zebrafish gastrulation, an increase in Mmp14 activity was observed in *vangl2* mutant embryos [100]. Thus, the decreased FBMN filopodial stability observed when Vangl2 is absent in the migratory environment could be due to decreased extracellular matrix.

Which cells in the environment of FBMNs are the source of PCP cues for filopodial dynamics and migration? We have shown that disruption of PCP signaling in the r4 environment prevents FBMN migration, demonstrating that PCP signaling is required to initiate directional migration. It was recently reported that Vangl2 expression even in a single cell in the r4 floorplate is sufficient to rescue FBMN migration in a *vangl2* mutant [55]. In contrast, our results show that floorplate Vangl2 is neither required nor sufficient for FBMN migration. Neither the widespread presence of GFP-Vangl2 expressing cells or of wild type donor-derived cells in the floorplate of *vangl2* mutants rescued the migration of *vang2* mutant FBMNs. The rescue of FBMN migration observed by Sittaramane et al. (2013) may have been due to undetected early expression of Vangl2 outside of the floorplate driven by the Gal4 genetrap line used in their experiments [101, 102].

We did, however, note that the presence of wild type cells in the floorplate could partially rescue the migration of wild type FBMNs in an otherwise *vangl2* mutant embryo. This suggests that bidirectional PCP signaling between the planar-polarized floorplate and the FBMNs can promote migration. However this rescue was incomplete, indicating that other planar polarized cells in the r4 environment normally contribute to the pro-migratory environment. Consistent with this hypothesis, we found that disrupting the planar polarization of the floorplate alone in both fish and mouse embryos was insufficient to prevent FBMN migration (Fig 3 and S4 Fig). We conclude that the planar polarization of the entire r4 environment surrounding the FBMNs is required to effectively initiate migration. We were unable to confirm this by rescuing FBMN migration in a *vangl2* mutant with r4-restricted expression of GFP-Vangl2, likely because the over-expression of PCP components disrupts planar polarity as efficiently as their loss [28, 31, 103, 104].

Our study provides new insights into the role of the planar cell polarity pathway in neuronal migration by identifying when and where PCP signaling is required and how it affects the dynamic cell behaviors of migrating neurons *in vivo*. Our data suggests that a planar polarized hindbrain rhombomere 4 neuroepithelium serves to promote FBMN migration through the selective stabilization and destabilization of FBMN filopodia using conserved intra- and intercellular interactions between the PCP components Vangl2 and Fzd3a. Whether neuroepithelial planar polarity directs posterior migration or simply enables it, and through what effectors PCP signaling regulates filopodial dynamics *in vivo* are important questions to be answered in future work.

## Methods

### Ethics statement

Experiments using animals were performed under the Fred Hutchinson Cancer Research Center Institutional Animal Care and Use Committee protocols #1392 (zebrafish, approved on 3/31/2015) and #50857 (mice, approved 4/1/2015). The Fred Hutchinson Cancer Research Center Institutional Animal Care and Use Committee (IACUC) follow the guidelines of the Office of Laboratory Animal Welfare and set its policies according to The Guide for the Care and Use of Laboratory Animals. Fred Hutchinson Cancer Research Center maintains full accreditation from the Association for Assessment and Accreditation of Laboratory Animal Care (AAALAC) and has letters of assurance on file with OLAW. The IACUC routinely evaluates the Fred Hutchinson animal facilities and programs to assure compliance with federal, state, local, and institution laws, regulations, and policies. The OLAW Assurance number is A3226-01.

### Zebrafish lines and maintenance

Zebrafish (*Danio rerio*) were raised at the Fred Hutchinson Cancer Research Center, and animal care and experiments were approved by the Institutional Animal Care and Use Committee. All animals were maintained according to standard procedures [105] and staged as previously described [106]. All mutant lines used were previously described and are registered at The Zebrafish International Resource Center (ZIRC): *fzd3a*^*rw689*^ (*olt*^*rw689*^) [49], *prickle1b*^*fh122*^ [50], and *vangl2*^*m209*^ (*tri*^*m209*^) [31]. Previously described transgenic lines used were as follows: *Tg(isl1*:*GFP)rw0* [56], *Tg(isl1CREST-hsp70l*:*mRFP)fh1* [67], *TgBAC(hoxb1a*:*RFP)fh3* [67], *Tg(egr2b*:*KalTA4)* [63] and *Tg(hoxb1a(β-globin)*:*Gal4VP16)um60* [64].

### Cloning and transgenic line generation

The following transgenic lines were generated for this study: *Tg(shh*:*Gal4VP16)fh445*, *Tg(isl1*:*Gal4VP16)fh452*, *Tg(isl1-hsp70*:*mTFP)fh350*, *Tg(isl-hsp70*:*dvl-DEP-GFP)fh444*, *Tg(10XUAS*:*xdd1-GFP)fh446*, *Tg(10XUAS*:*fzdΔC-GFP)fh447* and *Tg(10XUAS*:*GFP-vangl2)fh453*. The Gal4VP16 sequence was obtained from the Nonet Lab (http://pcg.wustl.edu/nonetlab/ResourcesF/Zebrafish.html) and the 10XUAS plasmid was obtained from the Tol2 kit (http://tol2kit.genetics.utah.edu/index.php/List_of_entry_and_destination_vectors) [107]. The mTFP construct was obtained from Alleleustrious, Inc (Cat# ABP-FP-TFA1000).

To generate *Tg(shh*:*Gal4VP16)fh445*, the ar-B enhancer element of zebrafish *sonic hedgehog (shh)* [108, 109] was amplified from a plasmid (gift from Uwe Strähle). For the Gal4 lines, the *shh* and *isl1* enhancers were inserted upstream of the *gata2* minimal promoter element [110]. The Xdd1 and full-length *Xenopus* Dvl are described in Sokol et al. (1996) [25]. Transgenic elements were cloned using the Gateway (Life Technologies) system using the primer sequences listed in S1 Table. Final DNA constructs were assembled in the pDESTpBHR4R3 plasmid (gift from the Brockerhoff Lab) or the CG5 Tol2 expression vector [107]. Transgenic embryos were generated by Tol2 transposase RNA co-injection with each plasmid at the single cell stage [111].

### Mouse lines and husbandry

All mice were maintained at Fred Hutchinson Cancer Research Center under Institutional Animal Care and Use Committee approved guidelines. For general colony maintenance, all mouse lines were crossed into the C57BL/6J background (The Jackson Laboratory strain 00064). The *Vangl2*^Loxp^ and *Vangl2*^ΔTM^ lines were a gift from the Deans laboratory [61], the *Isl1Cre* (*Isl*^*tm1(cre)Sev*^) line was a gift from the Evans laboratory [62] and the Shh:gfp-cre *(Shh*^*tm1(EGFP/cre)Cjt*^) line was purchased from The Jackson Laboratory (strain 005622).

### Cell transplantation

Chimeric embryos were generated by transplantation at the blastula or gastrula stage as previously described [51, 112]. To track transplanted cells, donor embryos carrying the *Tg(isl1*:*GFP)rw0*, *Tg(isl1*:*mRFP)fh1* or *Tg(isl1*:*mTFP)fh350* transgene were injected with 1% cascade blue-dextran or rhodamine-dextran (for live imaging) and 1% biotin-dextran (for imaging after fixation) (10,000 mw, Life Technologies). Host embryos were then processed and imaged for all donor-derived cells, donor-derived FBMNs or floorplate cells, and host FBMNs. Host and donor embryo genotypes were identified either by observing body axis elongation defects (for *vangl2* mutant hosts), by examining FBMN location at 48 hpf or by genotyping (for *fzd3a* mutant hosts).

For transplantation of post-mitotic FBMNs, cascade blue-dextran labeled donor embryos and unlabeled host embryos were mounted in agar on coverslips at the 15-somite stage. The head of each animal was exposed by careful removal of agar with insect pins, and a hole was cut in the skin overlying the forebrain to enable entry of a thin (10 μm diameter) transplant pipette. Pre-migratory FBMNs (visualized by *isl1*:*GFP* or *isl1*:*mRFP* expression) were removed from r4 of a donor embryo and transplanted into r4 of a host embryo using a Zeiss AxioSkop fixed-stage microscope fitted with a 40X long working-distance water-immersion (“dipping”) lens. During this process some non-*isl1*:*GFP/mRFP*-expressing neuroepithelial progenitor cells were inevitably co-transplanted but these usually died shortly after transplantation; any surviving donor-derived cells that were not FBMNs were detected by the presence of the cascade blue dye. Due to the disruptive approach, which removes nascent axons and other processes, even wild type FBMNs transplanted into a wild type environment do not migrate as well as FBMNs transplanted at earlier stages.

### Whole-mount immunohistochemistry

Anesthetized zebrafish embryos were fixed in 2% trichloroacetic (TCA) acid for 3 hours or 4% paraformaldehyde (PFA)/ 4% sucrose in PBS for 1 hour at room temperature. Dissected mouse hindbrains were fixed in 4% paraformaldehyde (PFA)/ 4% sucrose in PBS overnight at 4°C and permeablized in PBS + 1% TritonX100. Fixed tissue was washed in PBS + 0.5% TritonX100 followed by standard blocking and antibody incubations. Following staining, brain tissue was dissected, cleared step-wise in a 25%, 50%, 75% glycerol series and mounted for confocal imaging. The following antibodies were used: rabbit anti-zebrafish Vangl2 (1:250, Anaspec Cat# AS-55659), mouse anti-islet1 39.4D5 (1:10 for zebrafish tissue and 1:100 for mouse tissue, Developmental Studies Hybridoma Bank); chicken anti-GFP (1:500, Abcam Cat# ab13970); rabbit anti-ZO-1 (1:1000, Zymed Cat# 61–7300); mouse anti-Cc2d2a (1:100, [113]); rabbit anti-RFP (1:1000, Abcam Cat# ab62341). For analysis of chimeric embryos after fixation, host embryos were additionally stained with a fluorescently conjugated streptavidin (Life Technologies Cat# S32351) to enhance the detection of Biotin-Dextran-containing donor-derived cells.

### Primary cell culture

Primary cultures of FBMNs were prepared from 24 hour post fertilization *Tg(isl1*:*mTFP)*; *Tg(hoxb1a*:*RFP)* embryos. The hindbrains of embryos were micro-dissected and dissociated as previously reported [114]. Cells were plated on a chambered coverglass (Sigma Z734756) coated with 5μg/mL poly-D-lysine (Sigma L8021) and 5μg/mL laminin (Sigma L2020) at a density of 4–5 hindbrains per 1.7 cm^2^. FBMNs were distinguished from other *Tg(isl1*:*mTFP)*-expressing hindbrain motor neurons by virtue of *Tg(hoxb1a*:*RFP)* expression, which is restricted to hindbrain r4 and r4-derived neurons. Live imaging of explanted neurons was performed 5 hours after plating.

### Imaging and data analysis

Imaging was performed using a Zeiss 700 confocal microscope or a Zeiss spinning disc microscope with a QuantEM EMCCD camera for live time-lapse imaging. For timelapse imaging, Z-stack images at 1μm steps were captured every 30 seconds for 15 minutes for *in vivo* time-lapse images and every 5 seconds for 10 minutes for cultured neurons. Filopodia were defined as long thin protrusions, less than 0.2 μm in diameter and more than 0.75 μm in length, measured from the cell body margin to the protrusion tip. *In vivo* filopodia lengths, lifetimes and fluorescent intensities of mRFP and GFP-Vangl2 were quantified using Zeiss Zen 2012 software. For cultured neurons, filopodium quantification was performed semi-automatically using Imaris FilamentTracer software (http://www.bitplane.com/imaris/filamenttracer). Mean anti-Vangl2 fluorescent intensity for all cell membranes were measured in user-drawn regions of interest using Zeiss Zen 2011 software or ImageJ's "Plot Profile" tool. Anterior/posterior GFP-Vangl2 fluorescent intensity ratios for each cell were normalized by dividing this value by the anterior/posterior ZO-1 fluorescent intensity ratio. Graphs were generated and statistics were computed using GraphPad Prism software. All statistical analyses were performed using a 95% confidence interval. In most cases significance was determined using an unpaired, two-tail t-test with Welch’s correction. For the anti-Vangl2 staining quantification significance was determined using a paired two-tail t-test with Welch’s correction. Differences in FBMN distributions were analyzed using a Chi-square test where the distribution of FBMNs in control animals served as the expected frequencies or null hypothesis to determine if the observed frequencies were significantly different. Circular plots were generated using Oriana 4 software. Figure images were created using Adobe Photoshop and Adobe Illustrator.

## Supporting Information

S1 Fig Fzd3a has a cell-autonomous function in FBMN migration. (A-B) Live confocal images of 48 hpf chimeric embryos with anterior to the top. Transplant conditions are indicated as donor→host. Pk1bMO host embryos were used because they have normal neuroepithelial planar polarity but unmigrated FBMNs; this prevents donor-derived FBMNs from being carried to r6 by migrating host neurons in a PCP-independent manner. Cascade blue-dextran marks all donor-derived cells (blue), *Tg(isl1*:*mRFP)* marks host FBMNs (magenta) and *Tg(isl1*:*GFP)* marks donor-derived FBMNs (green). Histograms on the right indicate the percent of donor-derived FBMNs at 48 hpf that failed to migrate (rhombomere (r)4), partially migrated (r5) or fully migrated (r6) and numbers indicate the number of FBMNs represented in each bar. N indicates the number of chimeric embryos and n indicates the number of FBMNs scored in each condition. Brackets indicate rhombomere positon. Scale bar: 50 μm.
(TIF)

S2 Fig Post-mitotic FBMNs require PCP signaling for migration. (A) Live confocal image showing the dorsal view of a *pk1b* mutant embryo hindbrain at 48 hpf after transplantation of post-mitotic FBMNs from a wild type donor. Cascade blue-dextran marks all donor-derived cells (blue), *Tg(isl1*:*GFP)* marks host FBMNs (green) and *Tg(isl1*:*mRFP)* marks donor-derived FBMNs (magenta). (B) Histogram indicates the percent of donor-derived FBMNs at 48 hpf that failed to migrate, (rhombomere (r)4), partially migrated (r5) or fully migrated (r6) and numbers indicate the number of FBMNs represented in each bar. White arrows indicate migrated donor derived FBMNs. While post-mitotic FBMNs in general migrate poorly after being transplanted, they do sometimes migrate in WT and *pk1b* mutant hosts but never in *vangl2* mutant hosts (see Fig 2). Brackets indicate rhombomere positon. Scale bar: 50μm.
(TIF)

S3 Fig PCP-DN expression in the floorplate disrupts planar polarity. (A-C) *Tg(shh*:*Gal4)* driven expression of *Tg(UAS*:*Kaede)* in the notochord and floorplate of a 14 hpf (A) 24 hpf embryo (B) and a 48 hpf embryo (C). Anterior is to the left. Images are live lateral views in A-C and live dorsal views at the hindbrain level, A’,B’. (D-F) Confocal images showing floorplate planar polarity of the anterior spinal cord in 48 hpf zebrafish embryos. Anterior is to the top. Anti-ZO-1 marks subapical tight junctions (white), anti-Cc2d2a marks the basal bodies of the primary cilia (magenta, arrows), and anti-GFP indicates dominant negative protein expression (green). Scale bar: 10μm. Whereas basal bodies are localized toward the posterior membrane in wild type embryos (D), this polarity is disrupted in floorplate cells expressing Xdd1-GFP (E) or Fzd3aΔC-GFP (F) (arrows in E’ and F’). (G) Schematic of the method used to quantify floorplate planar polarity. Total cell length (x) is measured as the distance between the anterior and posterior membranes (white) at the level of the basal body (magenta). Basal body position (y) is measured as the distance between the anterior membrane and the basal body. Cellular planar polarity is quantified as the ratio of x/y. (H) Quantitation of average basal body position in the floor plate of 48 hpf embryos. Each data point represents the mean basal body position for all cells quantitated in a single embryo. WT: N = 34 embryos, 411 cells; Xdd1-GFP: N = 14 embryos, 207 expressing cells; FzdΔC-GFP: N = 29 embryos, 484 expressing cells; *vangl2*^*-/-*^: N = 10 embryos, 96 cells. Quantitation of floorplate polarity in *vangl2*^*-/-*^ embryos is included for comparison. Graph represents data as mean ± SD. **p<0.0001 compared to wild-type control.
(TIF)

S4 Fig Vangl2 is not required in the mouse floorplate for FBMN migration. (A-B) Dorsal view of E13.5 mouse hindbrains with FBMNs (magenta) labeled with anti-Isl1 staining. Dotted lines indicate length of facial motor nucleus. To improve the chances that a Cre-expressing cell will have a biallelic deletion of Vangl2, in these experiments we used the *Vangl2* ^*ΔTM*^ null allele, which we discovered belatedly to cause a mild FBMN migration defect in compound heterozygotes with the floxed *Vangl2*^*LoxP*^ allele. Nevertheless, deleting the floxed allele with *Shh*^*Cre*^ did not enhance the partial migration defect in *Vangl2*^*LoxP/ΔTM*^ controls. For the experiments using *Isl1Cre* shown in Fig 1 we did not use the *Vangl2* ^*ΔTM*^ allele. (A) FBMNs in a *Vangl2*^*LoxP/ΔTM*^ control embryo. N = 6 embryos. (B) FBMNs in *Vangl2*^*LoxP/ ΔTM*^;*Shh*^*Cre*^ embryo. Addition of *Shh*^*Cre*^ does not further disrupt FBMN migration. N = 4 embryos. (C) Quantitation of FBMN migration stream length in *Vangl2*^*LoxP/ΔTM*^ control embryos and *Vangl2*^*LoxP/ ΔTM*^;*Shh*^*Cre*^ embryos. Scale bar: 100μm
(TIF)

S5 Fig Specificity of the anti-Vangl2 antibody. (A-B) Dorsal view of wild type (A) and *vangl2* mutant (B) 24 hpf neural tubes immunostained with anti-Vangl2-NT (green). The neuroepithelial membrane staining visible in wild type is absent in the mutant. (C) Western blot analysis of whole embryo lysates with anti-Vangl2 antibody. Anti-alpha-tubulin was used as a loading control. Zebrafish Vangl2 is expected to run at approximately 60kDa. For the anti-Vangl2 blot there is a band that is present in the wild type and absent in the *vangl2* mutant, see asterisk.
(TIF)

S6 Fig Migrating FBMNs display polarized protrusions that fail to polarize in *vangl2* mutants. (A,C,E) Representative frames of mTFP expressing FBMNs from time-lapse images taken at 24 hpf to 32 hpf. (B,D,F) Each raw data point for protrusion angle is plotted on the circular graph below. Each division is 10 degrees. A, anterior. P, posterior. M, medial. L, lateral. Filopodia are radial in wild type FBMNs prior to exiting r4 (A,B, N = 3 embryos, 5 neurons, 28 filopodia) and become polarized to the posterior side of the cell during migration (C,D, N = 8 embryos, 10 neurons (6 in r5 and 4 in r6), 52 filopodia). FBMN protrusions fail to polarize in *vangl2* mutants (E,F, N = 5 embryos, 7 neurons, 61 protrusions). Scale bar: 16μm.
(TIF)

S7 Fig Donor-derived FBMNs used to quantitate filopodial dynamics were in a genetically chimeric environment. (A-E) Live confocal images of donor-derived FBMNs (green) and all other nearby donor-derived cells (magenta). Transplant conditions are indicated on as donor→host as in Fig 5. Rhodamine dextran marks all donor-derived cells (magenta), *Tg(isl1*:*mTFP)* marks donor-derived FBMNs (green). Anterior is to the top and medial is to the right. Scale bar: 5 μm.
(TIF)

S8 Fig Raw filopodial quantitation data. (A) Quantitation of filopodial lifetime for donor-derived FBMNs. Each data point represents one filopodium. The maximum filopodial lifetime (900 seconds) corresponds to the full length of the time-lapse. (B) Quantitation of maximum filopodial length for filopodia lasting longer than 90 seconds on donor-derived FBMNs. Each data point represents one filopodium.
(TIF)

S9 Fig The effect of PCP on protrusion dynamics is dependent on the migratory environment. (A) Method used to isolate and identify FBMNs in primary culture. Embryos used were *Tg(isl1*:*mTFP)*;*Tg(hoxb1a*:*RFP)* allowing for the differentiation between FBMNs and other branchiomotor neurons labeled by *Tg(isl1*:*mTFP)*. (B,C) Cultured *Tg(isl1*:*mTFP)*; *Tg(hoxb1a*:*RFP)* FBMNs from a wild type (B) and a *vangl2* mutant embryo (C). (B’,C’) Time-lapse spinning-disc confocal series of boxed region from B and C. (D) Quantitation of filopodial lifetime for cultured FBMNs. Each timelapse was 600 seconds total. p = 0.9044, n.s. (E) Quantitation of the maximum filopodial length for cultured FBMNs. p = 0.0856, n.s. Wild type: N = 8 neurons, 64 filopodia. *vangl2*^*-/-*^: N = 8 neurons, 61 filodpodia. Graphs represent data as mean ± SEM. Each data point is the average lifetime (D) or maximum length (E) for all the filopodia of one FBMN. Significance was determined using an unpaired, two-tail t-test with Welch’s correction.
(TIF)

S1 Movie Time-lapse of a GFP-Vangl2; *Tg(isl1*:*mRFP)* expressing FBMN. Note GFP-Vangl2 enrichment events preceding filopodia retraction. Movie is 4 frames per second (fps) with a 45 second time interval.
(AVI)

S2 Movie Time-lapse of LifeAct-GFP expressing FBMN. Movie is 4fps with a 13 second time interval.
(AVI)

S3 Movie Time-lapse of a *Tg(isl1*:*mTFP)* wild type FBMN in a wild type host. This and all subsequent movies are 4fps with 30 second time intervals.
(AVI)

S4 Movie Time-lapse of *Tg(isl1*:*mTFP) vangl2* mutant FBMNs in a *vangl2* mutant host. (AVI)

S5 Movie Time-lapse of a *Tg(isl1*:*mTFP) vangl2* mutant FBMN in a wild type host. (AVI)

S6 Movie Time-lapse of a *Tg(isl1*:*mTFP) fzd3a* mutant FBMN in a wild type host. (AVI)

S7 Movie Time-lapse of a *Tg(isl1*:*mTFP)* wild type FBMN in a *vangl2* mutant host. (AVI)

S8 Movie Time-lapse of a *Tg(isl1*:*mTFP)* wild type FBMNs in a *fzd3a* mutant host. (AVI)

S1 Table Primers used in the creation of transgenic constructs. (PDF)
