# Supplementary figures and images for: PCP Signaling between Migrating Neurons and their Planar-Polarized Neuroepithelial Environment Controls Filopodial Dynamics and Directional Migration
Source: PLoS Genet. 2016 Mar 18;12(3):e1005934. doi: 10.1371/journal.pgen.1005934 (PMC4798406; doi:10.1371/journal.pgen.1005934)

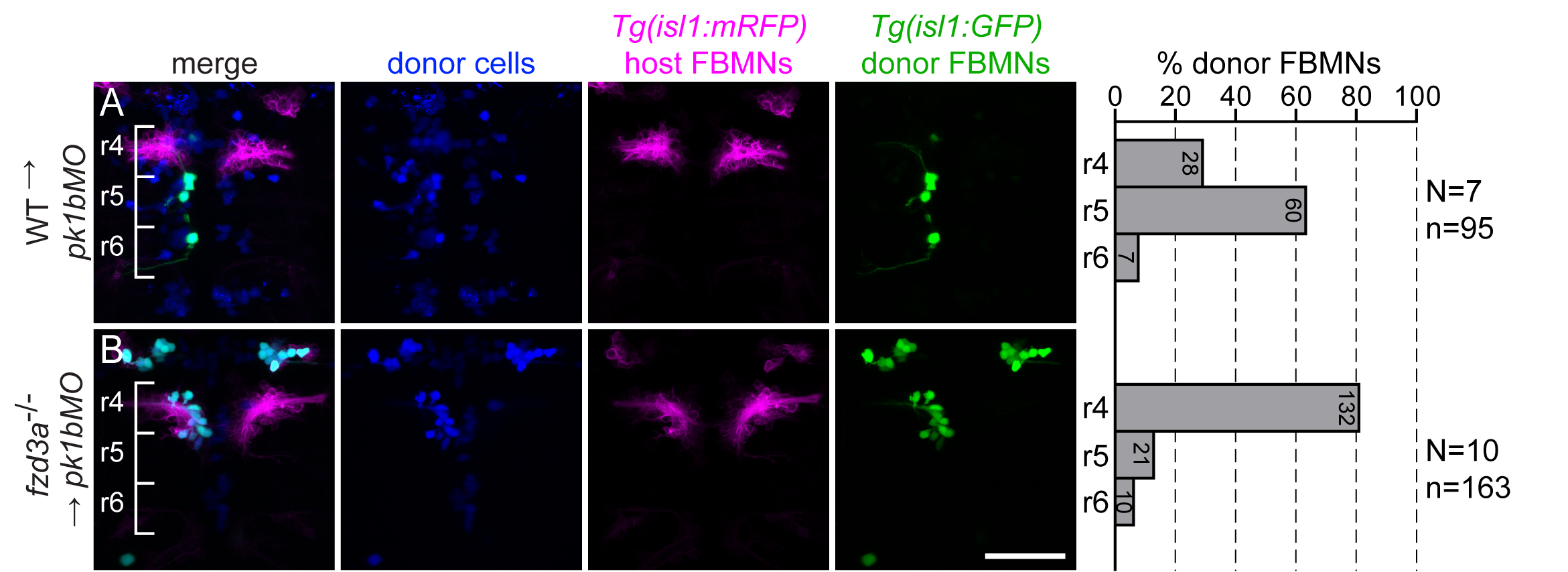

Supplement: S1 Fig — (A-B) Live confocal images of 48 hpf chimeric embryos with anterior to the top. Transplant conditions are indicated as donor→host. Pk1bMO host embryos were used because they have normal neuroepithelial planar polarity but unmigrated FBMNs; this prevents donor-derived FBMNs from being carried to r6 by migrating host neurons in a PCP-independent manner. Cascade blue-dextran marks all donor-derived cells (blue), Tg(isl1:mRFP) marks host FBMNs (magenta) and Tg(isl1:GFP) marks donor-derived FBMNs (green). Histograms on the right indicate the percent of donor-derived FBMNs at 48 hpf that failed to migrate (rhombomere (r)4), partially migrated (r5) or fully migrated (r6) and numbers indicate the number of FBMNs represented in each bar. N indicates the number of chimeric embryos and n indicates the number of FBMNs scored in each condition. Brackets indicate rhombomere positon. Scale bar: 50 μm. (TIF) [file pgen.1005934.s001.tif]

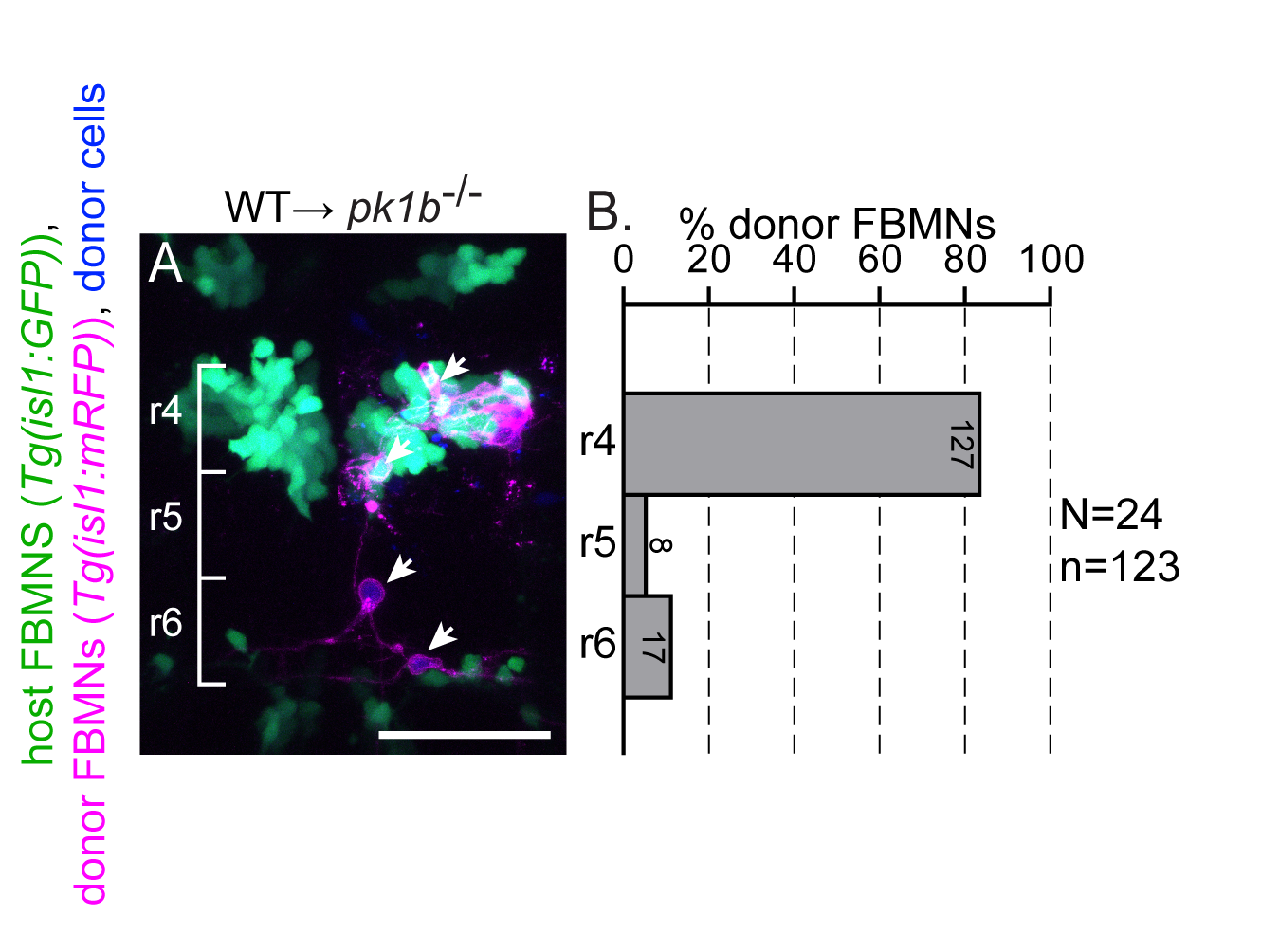

Supplement: S2 Fig — (A) Live confocal image showing the dorsal view of a pk1b mutant embryo hindbrain at 48 hpf after transplantation of post-mitotic FBMNs from a wild type donor. Cascade blue-dextran marks all donor-derived cells (blue), Tg(isl1:GFP) marks host FBMNs (green) and Tg(isl1:mRFP) marks donor-derived FBMNs (magenta). (B) Histogram indicates the percent of donor-derived FBMNs at 48 hpf that failed to migrate, (rhombomere (r)4), partially migrated (r5) or fully migrated (r6) and numbers indicate the number of FBMNs represented in each bar. White arrows indicate migrated donor derived FBMNs. While post-mitotic FBMNs in general migrate poorly after being transplanted, they do sometimes migrate in WT and pk1b mutant hosts but never in vangl2 mutant hosts (see Fig 2). Brackets indicate rhombomere positon. Scale bar: 50μm. (TIF) [file pgen.1005934.s002.tif]

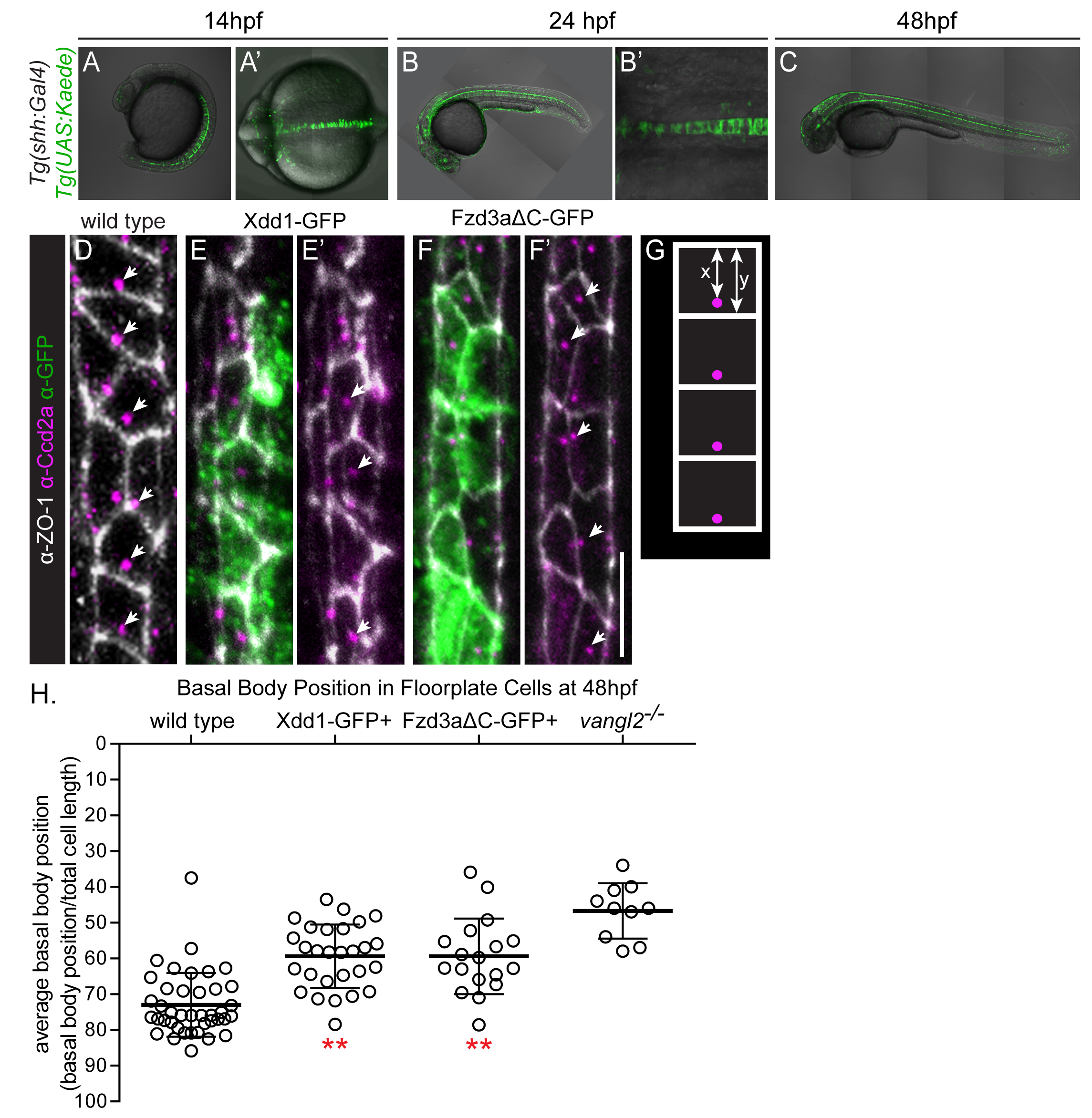

Supplement: S3 Fig — (A-C) Tg(shh:Gal4) driven expression of Tg(UAS:Kaede) in the notochord and floorplate of a 14 hpf (A) 24 hpf embryo (B) and a 48 hpf embryo (C). Anterior is to the left. Images are live lateral views in A-C and live dorsal views at the hindbrain level, A’,B’. (D-F) Confocal images showing floorplate planar polarity of the anterior spinal cord in 48 hpf zebrafish embryos. Anterior is to the top. Anti-ZO-1 marks subapical tight junctions (white), anti-Cc2d2a marks the basal bodies of the primary cilia (magenta, arrows), and anti-GFP indicates dominant negative protein expression (green). Scale bar: 10μm. Whereas basal bodies are localized toward the posterior membrane in wild type embryos (D), this polarity is disrupted in floorplate cells expressing Xdd1-GFP (E) or Fzd3aΔC-GFP (F) (arrows in E’ and F’). (G) Schematic of the method used to quantify floorplate planar polarity. Total cell length (x) is measured as the distance between the anterior and posterior membranes (white) at the level of the basal body (magenta). Basal body position (y) is measured as the distance between the anterior membrane and the basal body. Cellular planar polarity is quantified as the ratio of x/y. (H) Quantitation of average basal body position in the floor plate of 48 hpf embryos. Each data point represents the mean basal body position for all cells quantitated in a single embryo. WT: N = 34 embryos, 411 cells; Xdd1-GFP: N = 14 embryos, 207 expressing cells; FzdΔC-GFP: N = 29 embryos, 484 expressing cells; vangl2-/-: N = 10 embryos, 96 cells. Quantitation of floorplate polarity in vangl2-/- embryos is included for comparison. Graph represents data as mean ± SD. **p<0.0001 compared to wild-type control. (TIF) [file pgen.1005934.s003.tif]

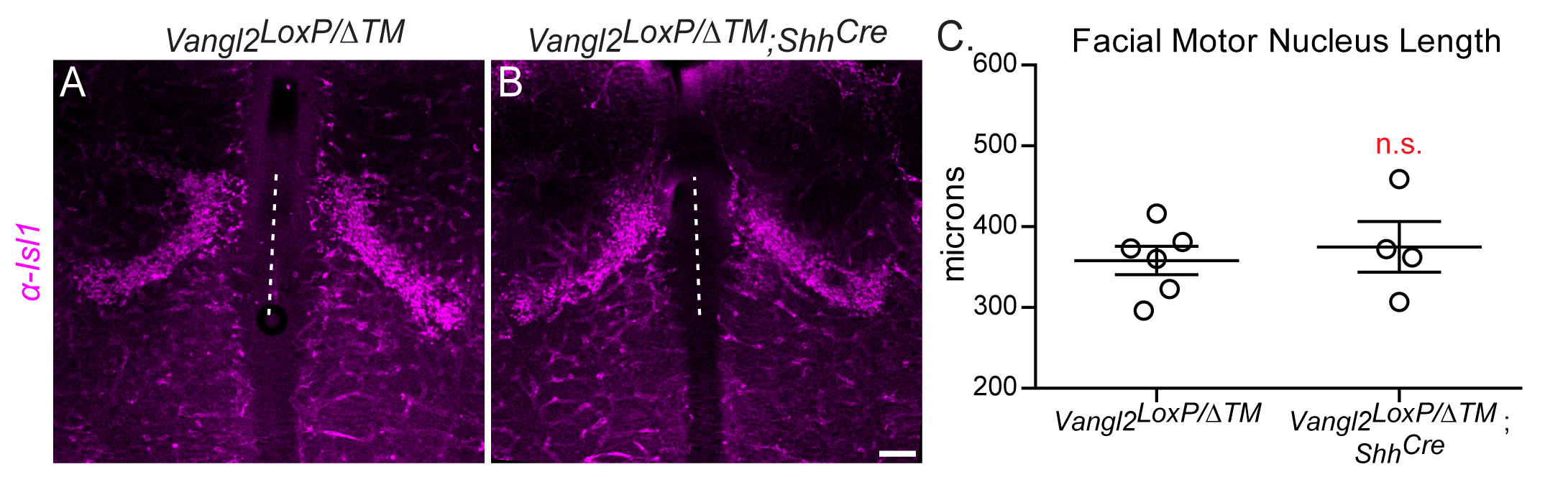

Supplement: S4 Fig — (A-B) Dorsal view of E13.5 mouse hindbrains with FBMNs (magenta) labeled with anti-Isl1 staining. Dotted lines indicate length of facial motor nucleus. To improve the chances that a Cre-expressing cell will have a biallelic deletion of Vangl2, in these experiments we used the Vangl2 ΔTM null allele, which we discovered belatedly to cause a mild FBMN migration defect in compound heterozygotes with the floxed Vangl2LoxP allele. Nevertheless, deleting the floxed allele with ShhCre did not enhance the partial migration defect in Vangl2LoxP/ΔTM controls. For the experiments using Isl1Cre shown in Fig 1 we did not use the Vangl2 ΔTM allele. (A) FBMNs in a Vangl2LoxP/ΔTM control embryo. N = 6 embryos. (B) FBMNs in Vangl2LoxP/ ΔTM;ShhCre embryo. Addition of ShhCre does not further disrupt FBMN migration. N = 4 embryos. (C) Quantitation of FBMN migration stream length in Vangl2LoxP/ΔTM control embryos and Vangl2LoxP/ ΔTM;ShhCre embryos. Scale bar: 100μm (TIF) [file pgen.1005934.s004.tif]

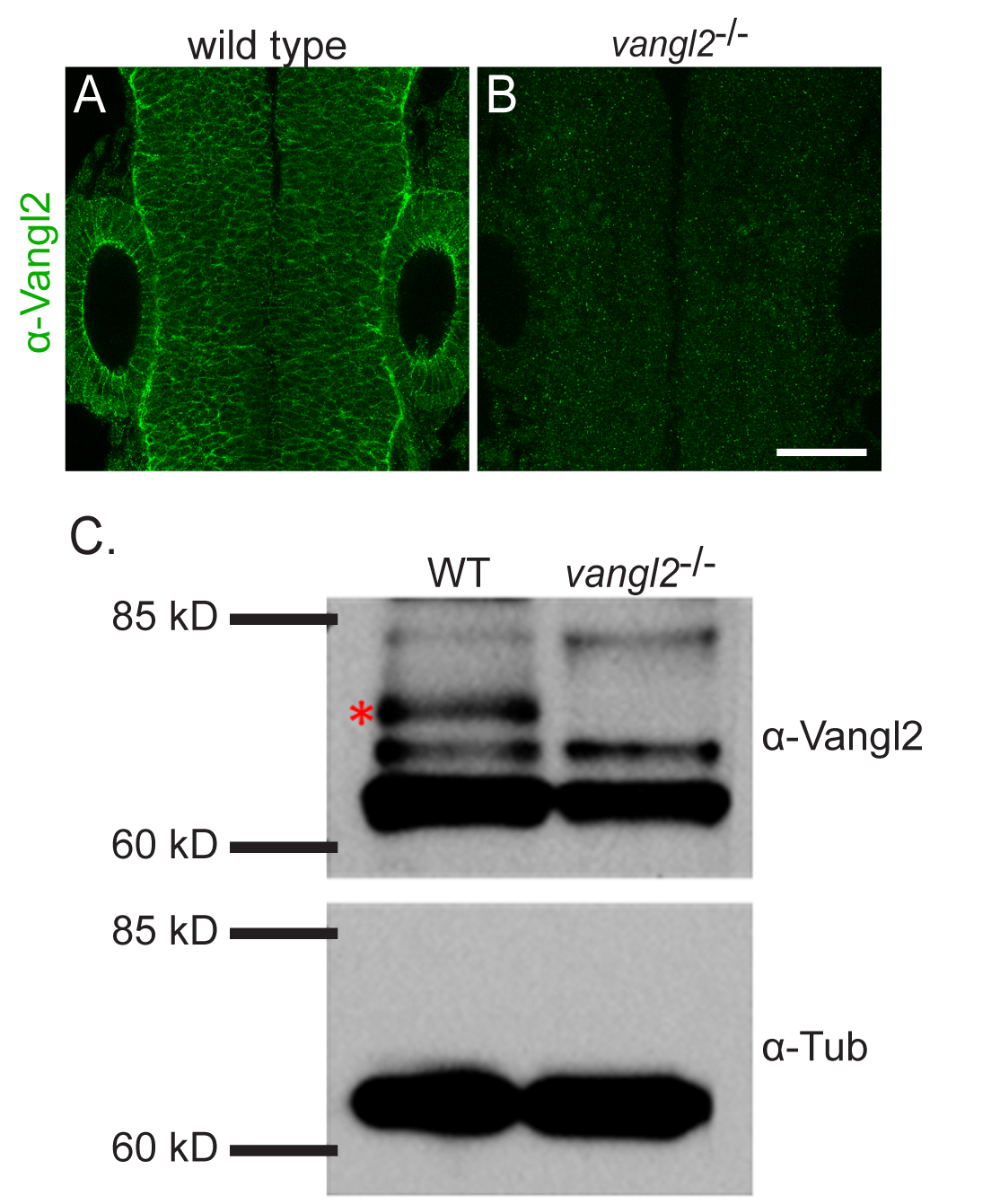

Supplement: S5 Fig — (A-B) Dorsal view of wild type (A) and vangl2 mutant (B) 24 hpf neural tubes immunostained with anti-Vangl2-NT (green). The neuroepithelial membrane staining visible in wild type is absent in the mutant. (C) Western blot analysis of whole embryo lysates with anti-Vangl2 antibody. Anti-alpha-tubulin was used as a loading control. Zebrafish Vangl2 is expected to run at approximately 60kDa. For the anti-Vangl2 blot there is a band that is present in the wild type and absent in the vangl2 mutant, see asterisk. (TIF) [file pgen.1005934.s005.tif]

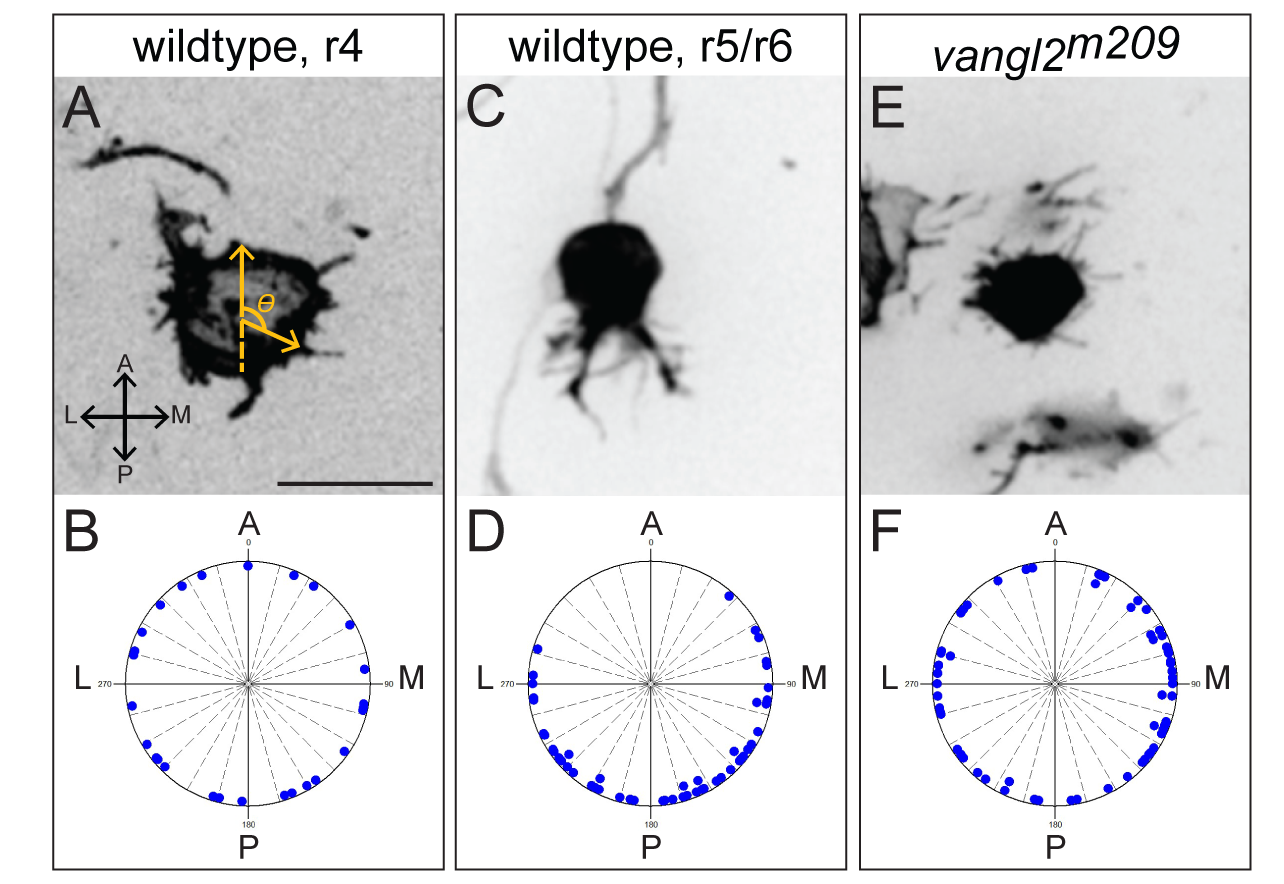

Supplement: S6 Fig — (A,C,E) Representative frames of mTFP expressing FBMNs from time-lapse images taken at 24 hpf to 32 hpf. (B,D,F) Each raw data point for protrusion angle is plotted on the circular graph below. Each division is 10 degrees. A, anterior. P, posterior. M, medial. L, lateral. Filopodia are radial in wild type FBMNs prior to exiting r4 (A,B, N = 3 embryos, 5 neurons, 28 filopodia) and become polarized to the posterior side of the cell during migration (C,D, N = 8 embryos, 10 neurons (6 in r5 and 4 in r6), 52 filopodia). FBMN protrusions fail to polarize in vangl2 mutants (E,F, N = 5 embryos, 7 neurons, 61 protrusions). Scale bar: 16μm. (TIF) [file pgen.1005934.s006.tif]

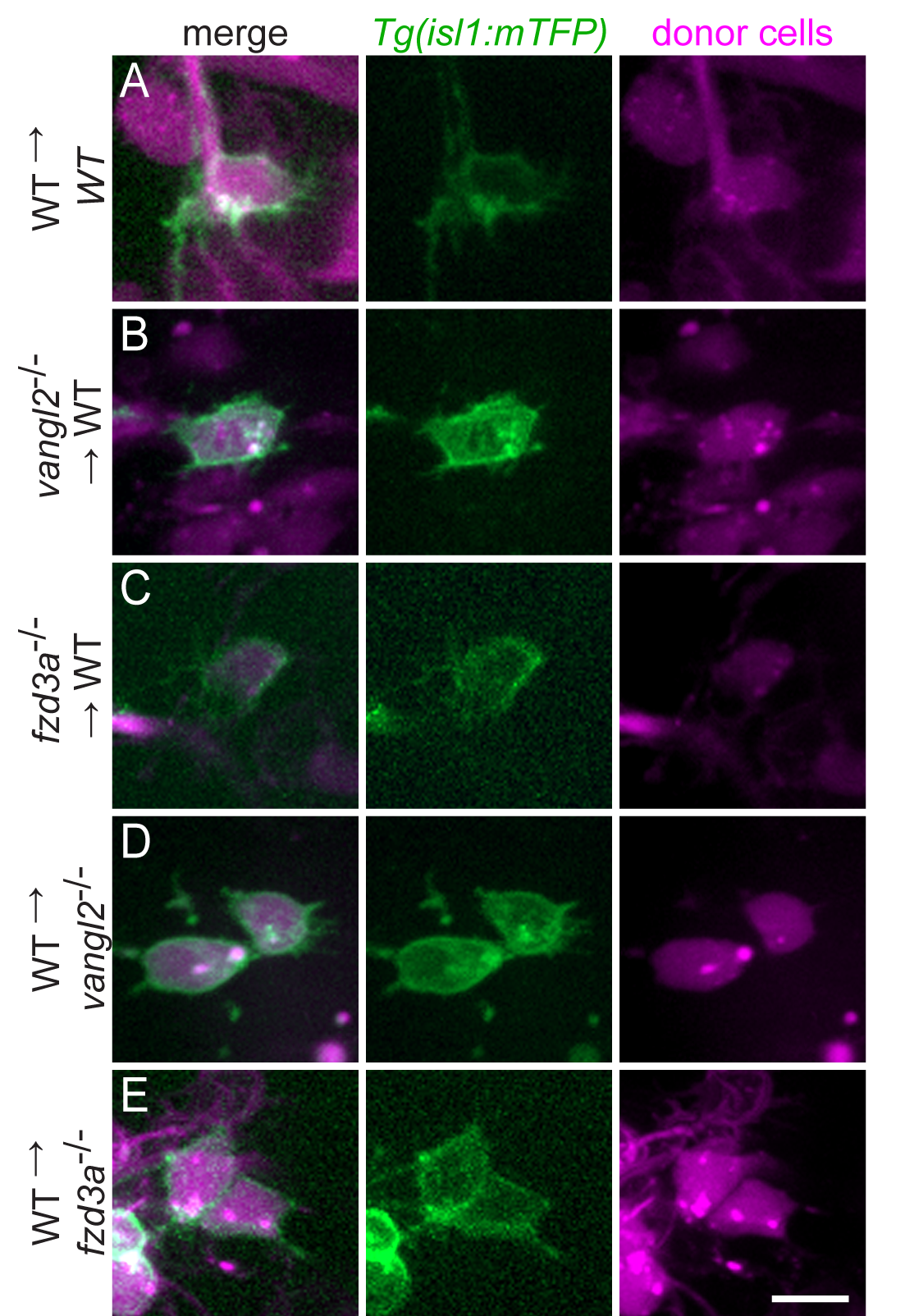

Supplement: S7 Fig — (A-E) Live confocal images of donor-derived FBMNs (green) and all other nearby donor-derived cells (magenta). Transplant conditions are indicated on as donor→host as in Fig 5. Rhodamine dextran marks all donor-derived cells (magenta), Tg(isl1:mTFP) marks donor-derived FBMNs (green). Anterior is to the top and medial is to the right. Scale bar: 5 μm. (TIF) [file pgen.1005934.s007.tif]

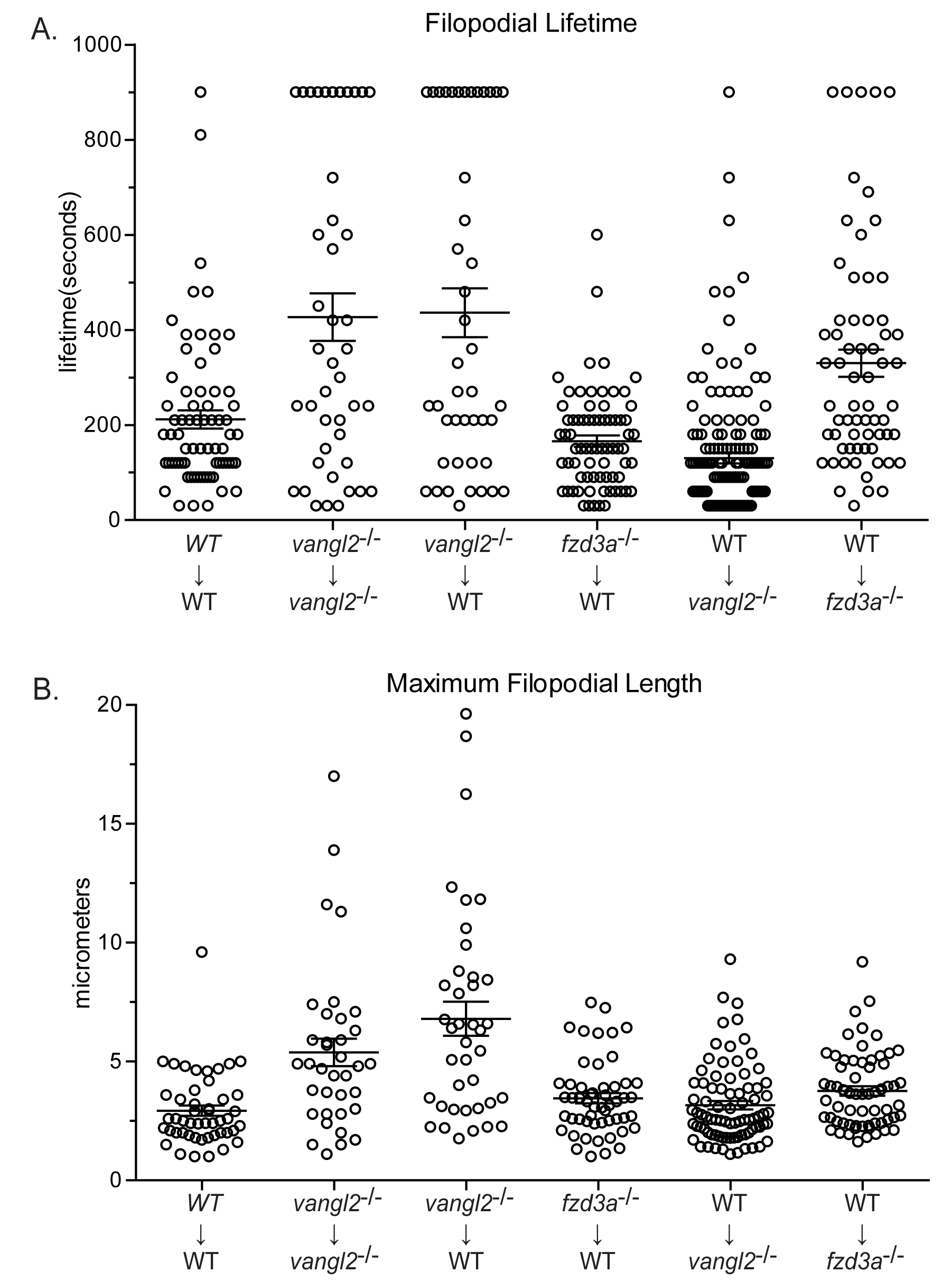

Supplement: S8 Fig — (A) Quantitation of filopodial lifetime for donor-derived FBMNs. Each data point represents one filopodium. The maximum filopodial lifetime (900 seconds) corresponds to the full length of the time-lapse. (B) Quantitation of maximum filopodial length for filopodia lasting longer than 90 seconds on donor-derived FBMNs. Each data point represents one filopodium. (TIF) [file pgen.1005934.s008.tif]

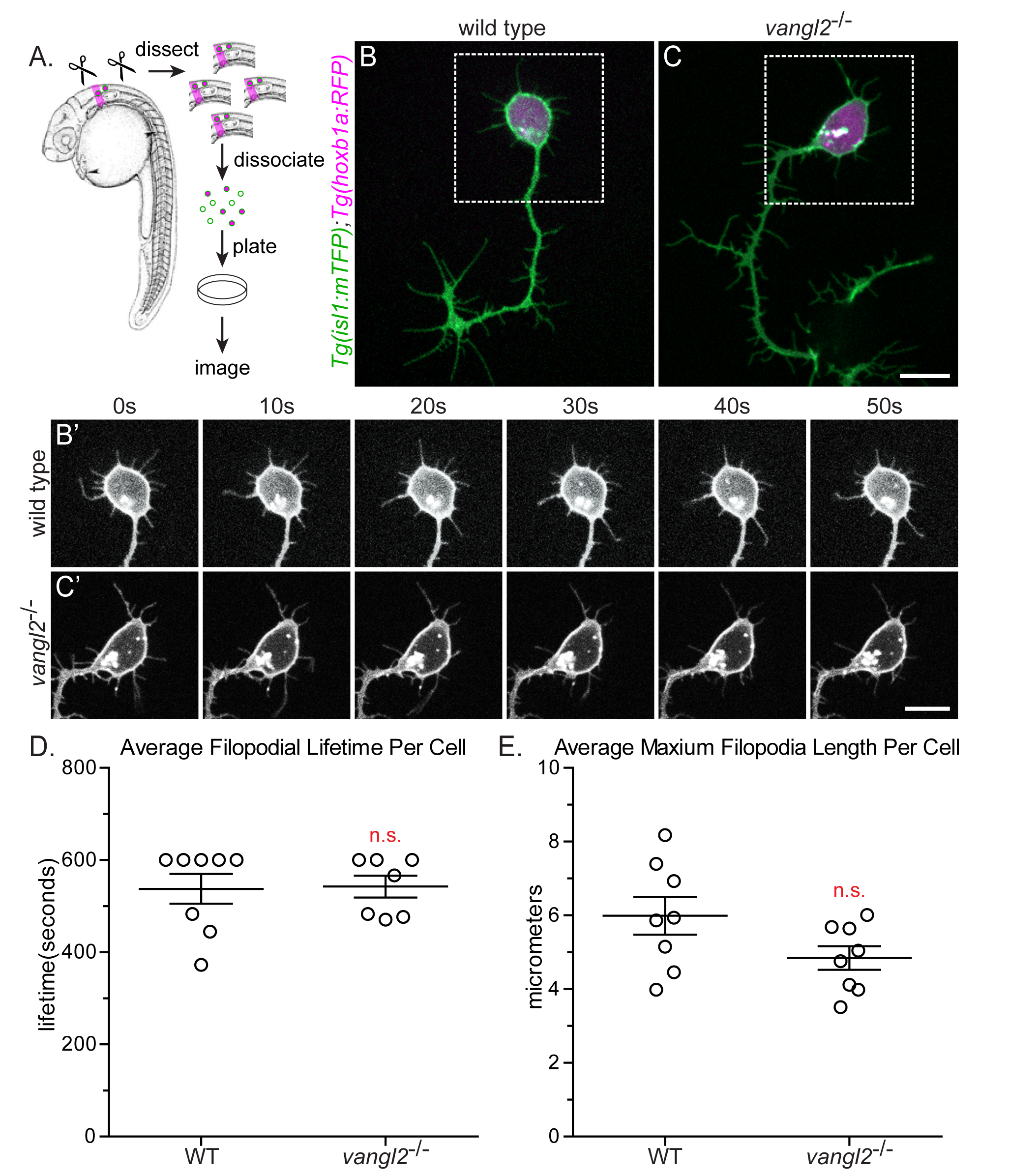

Supplement: S9 Fig — (A) Method used to isolate and identify FBMNs in primary culture. Embryos used were Tg(isl1:mTFP);Tg(hoxb1a:RFP) allowing for the differentiation between FBMNs and other branchiomotor neurons labeled by Tg(isl1:mTFP). (B,C) Cultured Tg(isl1:mTFP); Tg(hoxb1a:RFP) FBMNs from a wild type (B) and a vangl2 mutant embryo (C). (B’,C’) Time-lapse spinning-disc confocal series of boxed region from B and C. (D) Quantitation of filopodial lifetime for cultured FBMNs. Each timelapse was 600 seconds total. p = 0.9044, n.s. (E) Quantitation of the maximum filopodial length for cultured FBMNs. p = 0.0856, n.s. Wild type: N = 8 neurons, 64 filopodia. vangl2-/-: N = 8 neurons, 61 filodpodia. Graphs represent data as mean ± SEM. Each data point is the average lifetime (D) or maximum length (E) for all the filopodia of one FBMN. Significance was determined using an unpaired, two-tail t-test with Welch’s correction. (TIF) [file pgen.1005934.s009.tif]
