# Supplementary material for: PCP Signaling between Migrating Neurons and their Planar-Polarized Neuroepithelial Environment Controls Filopodial Dynamics and Directional Migration
Source: PLoS Genet. 2016 Mar 18;12(3):e1005934. doi: 10.1371/journal.pgen.1005934 (PMC4798406; doi:10.1371/journal.pgen.1005934)
Supplement: S1 Table — (PDF) [file pgen.1005934.s018.pdf]

| Construct | Forward Primer Sequence | Reverse Primer Sequence |
|---|---|---|
| *shh-ArD* | 5’-GGTACCGGCCTGCATGGC-3’ | 5’-ACTTGCACTAGTCTTCACGGCTCATCA-3’ |
| *xdd1* | 5'-ATGGCGGAGACTAAAGTGATTTAC-3' | 5’-TTACCTCATGACATCCACAAAGAACTC-3’ |
| *dvl-DEP* | 5’-ATGACCGTGGCCAAATGCTGGGAC-3’ | 5’-TTACCTCATGACATCCACAAAGAACTC-3’ |
| *gata2* | 5’-TGCAATAGGCCAAATCGACATTCA-3’ | 5’-CAAGTGTCCGCGCTTAGAAAATGC-3’ |
| *gal4VP16* | 5’-ATGAAGCTACTGTCTTCTATCGAA-3’ | 5’-CTACATATCCAGAGCGCCGTAGGG-3’ |
| *fzd3a∆C* | 5’-ATGGTTCTGCTTTGGGCTCTG-3’ | 5’-GGTCTTTTTACTCCCGACCCA-3’ |
| *vangl2* | 5'-ACCATGGATAACGAGTCGCAGTACTCA -3' | 5'-TCACACCGAGGTTTCCGACTGGAG-3' |
| *crest1(isl1)* | 5'-CTGAGTGGACCTGGCCACAGTCAA-3' | 5'-GTCTTGGAATGAAACAGACCA-3' |
| *fzd3a* | 5'-ACCATGGTTCTGCTTTGGGCTCTG-3' | 5'-TGCTTTGGTCGCGTCCTCCTC-3' |
